# Intelligent Thermo‐Self‐Limited Magnetothermia with Heat‐Triggered TERT Silencing for Precision Synergetic Cancer Therapy

**DOI:** 10.1002/advs.202519168

**Published:** 2026-01-30

**Authors:** Liang Zhang, Mingfu Gong, Tao Sun, Shilin Xiao, Yue Zhao, Xiaofeng Yang, Wansu Zhang, Chunyu Zhou, Xu Liu, Dong Zhang

**Affiliations:** ^1^ Department of Radiology Xinqiao Hospital Army Medical University Chongqing P. R. China

**Keywords:** Hsp70 promoter, in situ siRNA synthesis, self‐limited thermal, synergetic cancer therapy, ultra‐small iron oxide nanoparticles

## Abstract

The combination of hyperthermal therapy and gene therapy (GT) has emerged as a promising strategy for cancer treatment. However, the overheating damage and complex temperature control procedure in hyperthermal therapy, along with the limited precision of GT, greatly compromise the therapeutic effectiveness. In this study, a novel nanoplatform (IONP@H_1_THs) was rationally designed and fabricated by integrating ultra‐small iron oxide nanoparticles with functional DNAs, featuring self‐limiting magnetothermal performance and heat‐induced precision target gene editing for mild‐thermal therapy (MTT) and GT synergetic cancer treatment. Upon binding to telomerase reverse transcriptase (TERT) mRNA, the hairpin DNA within IONP@H_1_THs initiates self‐assembly through hairpin DNA–mediated dimer formation, thereby enhancing magnetothermal properties of IONP@H_1_THs in cancer cells specifically. Upon heating to a specified temperature of the assembled IONP@H_1_THs, the temperature‐sensitive double‐stranded DNA unwinds partially into single strands, resulting in reduced magnetic heating capacity and achieving a balance between heating and scattering, which enables self‐limited heating for MTT. Simultaneously, the mild thermal conditions activate transcription from the Hsp70 promoter, inducing in situ small interfering RNA synthesis for TERT gene knockdown to synergize with MTT‐mediated cancer treatment. Overall, this approach represents a promising strategy for tumor therapy by integrating thermo‐self‐limited MTT with heat‐triggered precise GT.

## Graphical abstract

Telomerase reverse transcriptase (TERT) mRNA induces DNA self‐assembly in cancer cells, boosting the nanoplatform's magnetothermal properties. This magnetothermia then activates the Hsp70 promoter, initiating siRNA synthesis to silence the TERT gene, enhancing cancer treatment. Specifically, the reversible nature of DNA‐mediated temperature‐sensitive assembly and disassembly demonstrates the self‐limiting magnetic thermal characteristic.

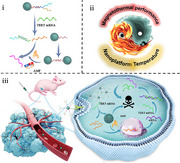

## 1 Introduction

Multifunctional therapeutic platforms that integrate various treatment modalities have garnered substantial interest due to their potential to provide effective cancer therapies [1, 2]. These platforms offer advantages such as enhanced anti‐tumor efficacy, minimized side effects, reduced drug dosages, and the ability to overcome multidrug resistance. In recent years, thermal therapy has gained considerable attention due to its capacity for precise local ablation of cancer cells in a time‐ and space‐controllable manner [3, 4]. However, cancer cells can develop thermotolerance through multiple mechanisms, such as the activation of heat shock proteins, inhibition of the mitochondrial apoptotic pathway, et al., potentially resulting in recurrence post‐treatment [5, 6]. Gene therapy (GT), an advanced medical technology designed to modulate gene expression for disease treatment, has the potential to synergize with thermal therapy by regulating the expression of genes associated with heat tolerance and other pertinent genetic factors, and thus combination constitutes a promising approach for cancer treatment and has attracted significant attention in recent decades [7, 8, 9].

Typically, hyper‐thermal stimulation at high local temperatures (> 50°C), which can induce necrosis of cancer cells directly [10]. Nevertheless, such high temperatures, along with the unintentional heating of non‐target tissues due to the non‐specific distribution of heat‐generating nanomaterials, can damage the normal tissues. Moreover, the direct necrosis of cancer cells will enhance the release of Toll‐like receptor‐related intratumoral molecules, triggering the production of immunosuppressive cytokines and facilitating tumor recurrence through immune escape, thereby reducing the therapeutic efficacy of hyper‐thermal therapy [11, 12, 13]. In contrast, mild thermal therapy (MTT), generally conducted at temperatures below 45°C, can modulate the activity of key proteins such as cyclins, kinases, and regulatory factors, leading to cellular thermal damage, disruption of the cell cycle, and apoptosis [14]. For nanomaterial‐based thermal therapy, the regulation of tumor temperature is generally achieved by adjusting external heating parameters, like the power of the laser [15, 16], the field strength and frequency of alternating magnetic field (AMF) [17]. However, these methods often require continuous monitoring of intratumoral temperature to dynamically adjust parameters, which poses significant challenges in practical applications, particularly for deep‐seated tumors [18]. A promising alternative is the development of nanoplatforms with self‐regulating heating capabilities to avoid overheating [19, 20].

Previous studies have shown that the magnetic induction heating performance of magnetic nanoparticles is strongly affected by their particle size and aggregation state. Ultra‐small iron oxide nanoparticle (IONP) exhibits negligible magnetic induction heating capacity, whereas suitable particle size increase or aggregation of ultra‐small IONP significantly enhances this property [21, 22, 23]. By designing a temperature‐sensitive, reversible nanoplatform capable of assembling and disassembling ultra‐small IONP could achieve self‐limited heating performance, preventing overheating of aggregated nanoparticles and facilitating effective MTT. However, MTT without adjuvant therapy fails to achieve complete tumor eradication as its therapeutic efficacy is significantly compromised by its suboptimal heat intensity [13, 24].

GT, an innovative medical technology, aims to treat diseases by modulating gene expression and is particularly relevant to cancers driven by genetic mutations or aberrant gene regulation, which can synergetic thermal therapy [25, 26, 27]. However, the relatively low delivery efficiency of therapeutic genes and the potential risk of off‐target effects in GT present substantial challenges to the practical implementation and wider adoption of this therapeutic strategy [28, 29]. Although various vectors, such as liposome [30, 31], polymer [32, 33], inorganic nanomaterial [34, 35], and engineered bacteria [36, 37], have recently shown notable advantages in gene delivery, including reduced immune activation, efficient tumor accumulation, and compatibility with combination therapies, achieving precise GT remains a considerable challenge [38, 39, 40]. Recently, stimuli‐responsive approaches have shown considerable promise for the controlled regulation of therapeutic genes [41], particularly through the in situ release and synthesis of therapeutic genes under external triggers, such as near‐infrared laser irradiation [34, 42], ultrasound [43, 44], and magnetic fields [45], to enhance precision. Among these, magnetic induction heating is especially attractive for thermal stimulation because it is not limited by tissue depth [46, 47]. Previous studies have shown that cells exposed to thermal stress can upregulate Hsp70 as a protective response. During this period, the transcriptional activity of the endogenous Hsp70 promoter is elevated, facilitating the regulation of cellular functions through the construction of an exogenous gene expression vector controlled by the Hsp70 promoter to drive the in situ synthesis of therapeutic genes [34, 48, 49]. This precise gene expression system of heat‐induced Hsp70 promoter transcription effectively bridges external stimuli with intracellular responses, significantly enhancing the specificity of GT and contributing to the advancement of novel therapeutic strategies in cancer treatment.

**SCHEME 1:**
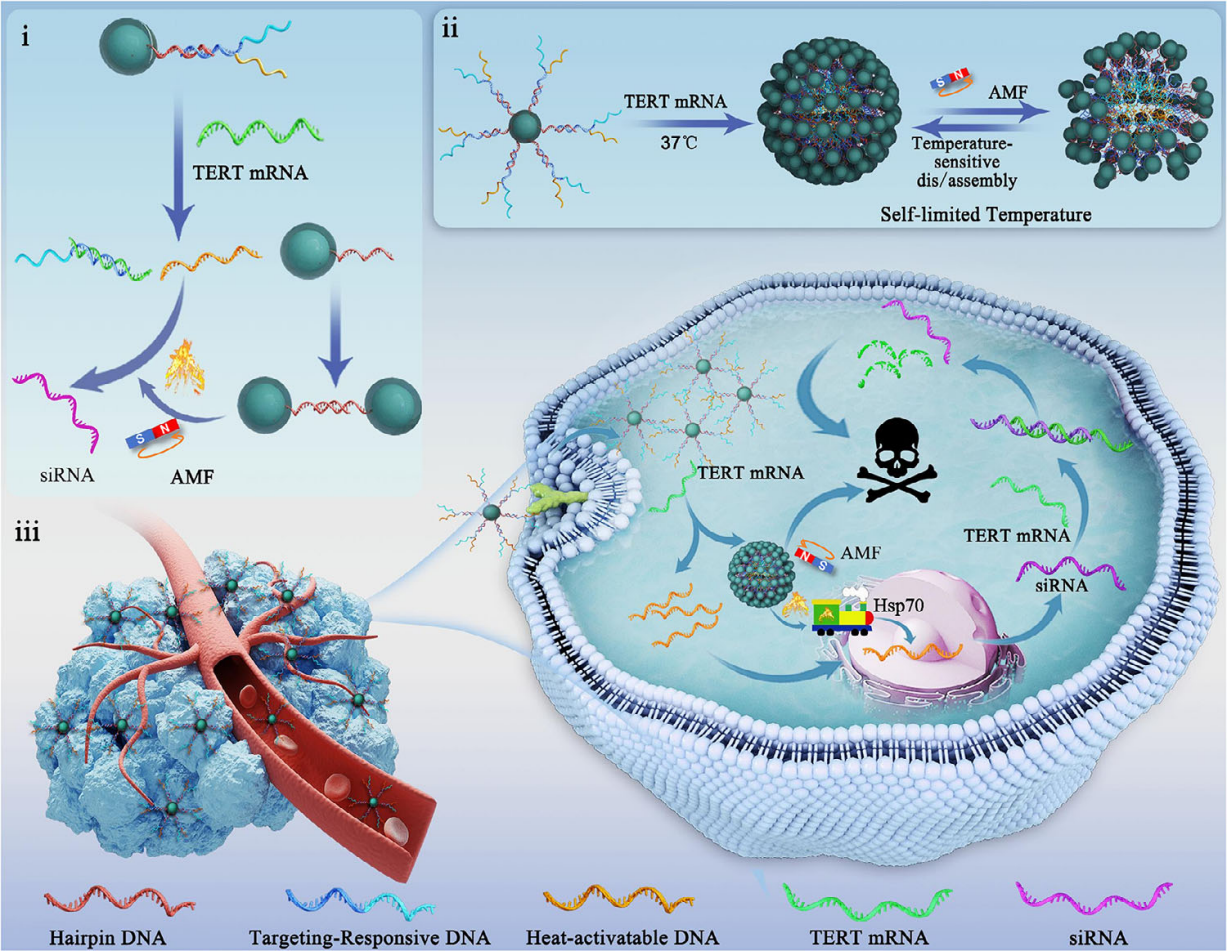
Schematic illustration of the TERT mRNA‐mediated self‐assembly, self‐limited magnetothermal, and heat‐triggered TERT silencing for precision synergistic cancer therapy. (i) The TERT mRNAs competitively bind with the responsive DNA strands on the nanoparticle surface, exposing the hairpin DNAs and triggering their self‐assembly, which enhances the magnetothermal effect of the nanoparticles and can be utilized for inducing the synthesis of small interfering RNA (siRNA). (ii) Mechanism of self‐limited heating by DNA‐mediated dynamic assembly and disassembly. At physiological temperature, extensive DNA hybridization leads to high assembly and strong magnetothermal effects. As the temperature increases, DNA denatures, reducing nanoparticle assembly and weakening the magnetothermal effect. (iii) Schematic illustration of magnetothermal and heat‐triggered gene editing for synergetic cancer therapy. TERT mRNA‐driven nanoparticle assembly boosts magnetothermal effects in cancer cells, activating the Hsp70 promoter under AMF to induce siRNA synthesis for synergetic cancer therapy with magnetothermia.

Telomerase reverse transcriptase (TERT), which serves as the catalytic subunit of the telomerase, plays an absolutely crucial role in the biological process of maintaining telomere length within cells. It is well‐established that TERT shows overexpression in approximately 85% to 90% of cancer cells across a wide range of malignancies, while typically inactive in most normal cells [50, 51, 52]. Suppression of TERT can lead to a remarkable inhibition of cell proliferation, induce apoptosis and accelerate senescence, and also enhance sensitivity to radiotherapy and chemotherapy, making it a promising therapeutic target [53]. In this study, we present a nanoplatform with self‐limiting magnetothermal property and heat‐induced TERT gene knockdown for precise GT and synergistic MTT in cancer therapy. Leveraging the reversible transition between double‐stranded DNA and single‐stranded DNA in response to temperature changes [54], we rationally designed a DNA‐mediated temperature‐sensitive self‐assembly and disassembly nanoplatform by integrating ultra‐small IONP with functional DNAs. This system enables self‐limiting MTT while simultaneously activating Hsp70 promoter–driven TERT knockdown, representing an effective synergetic strategy that integrates GT and MTT for precision cancer treatment (Scheme 1).

## 2 Results and Discussion

### 2.1 Synthesis and Characterization

**FIGURE 1:**
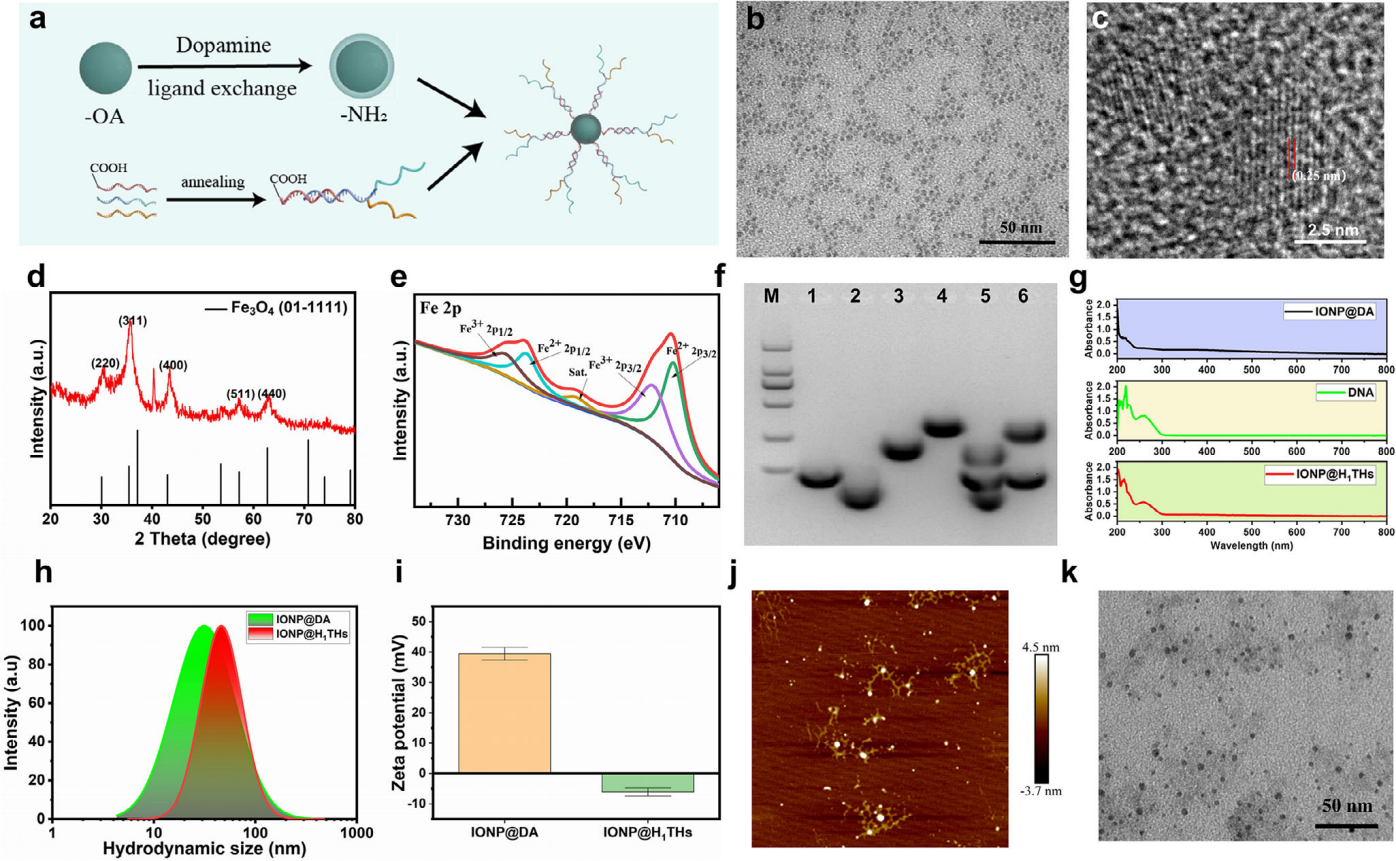
Preparation and characterization of IONP@H_1_THs. (a) Schematic illustration of the preparation of the nanoplatform. (b) TEM image of IONP@DA. (c) HRTEM image of IONP@DA. (d, e) X‐ray diffraction and X‐ray photoelectron spectroscopy analysis of IONP@DA, respectively. (f) Agarose gel electrophoresis analyzes the responsiveness of DNA hybrids. M, DNA marker; 1, TR‐DNA; 2, H_1_‐DNA; 3, Hs‐DNA; 4, H_1_THs‐DNA; 5, H_1_THs‐DNA + Target DNA; 6, H_1_THs‐DNA + Random DNA. (g) UV–vis spectroscopy analysis of IONP@H_1_THs. (h, i) Hydrodynamic size and Zeta potential of IONP@DA and IONP@H_1_THs (n=3), respectively. (j, k) Atomic force microscope and TEM images of IONP@H_1_THs, respectively.

The synthesis route for functional hybridized DNA‐modified ultra‐small IONP is illustrated in Figure 1a. The hydrophobic surface of oleic acid‐modified ultra‐small IONP (IONP‐OA) was replaced with a dopamine (DA) molecule through a ligand exchange method based on a previous study to obtain a hydrophilic nanoparticle (IONP@DA) [55]. The resulting IONP@DA was characterized using TEM and HRTEM. The TEM image shows that the nanoparticles possess a uniform spherical morphology (Figure 1b), and have a diameter of 3.2 ± 0.4 nm (Figure S1). HRTEM analysis (Figure 1c) revealed distinct lattice fringes with a spacing of 0.25 nm, corresponding to the (311) lattice plane of Fe_3_O_4_ [56]. The crystalline structure was further confirmed by X‐ray diffraction (Figure 1d), with sharp and intense peaks at (220), (311), (400), (511), and (440) matching the characteristic pattern of magnetite IONP [57]. X‐ray photoelectron spectroscopy (Figure 1e) was used to determine the iron valence states. The Fe 2p XPS spectrum exhibited a characteristic doublet at 705–715 and 720–730 eV, assigned to Fe 2p3/2 and Fe 2p1/2, respectively [57], confirming the successful synthesis of Fe_3_O_4_ nanoparticles.

To prepare the hybridized functional DNA (H_1_THs‐DNA), three distinct DNA strands, including a rationally designed Hairpin DNA (H_1_‐DNA), a Targeting‐Responsive DNA (TR‐DNA), and an Hsp70 promoter–controlled siRNA precursor (Hs‐DNA) were employed here. The specific nucleotide sequences were presented in Table S1. Initially, the three strands were annealed to facilitate the formation of hybrid structures. Subsequently, the hybridized DNA was analyzed and purified using agarose gel electrophoresis. The responsiveness of H_1_THs‐DNA was evaluated by introducing either the target TERT coding sequence or a random control DNA, followed by agarose gel electrophoresis. As shown in Figure 1f, TR‐DNA, H_1_‐DNA, and Hs‐DNA each exhibited distinct bands, all migrating faster than the hybridized DNA. Upon addition of the target DNA fragment (the same sequence as TERT mRNA), the hybridized DNA band disappeared, and three new bands corresponding to the component strands appeared. In contrast, the introduction of the random control DNA did not alter the hybridized DNA band, although a new band corresponding to the control DNA was detected. These findings confirm that the TERT coding sequence effectively triggers the disassembly of the hybridized DNA.

Finally, H_1_THs‐DNA was conjugated to ultra‐small IONP@DA via –NH_2_ and –COOH coupling using EDC/NHS chemistry to obtain a functional nanoplatform (IONP@H_1_THs). Successful DNA modification was confirmed by UV–vis spectroscopy (Figure 1g), which revealed a distinct absorption peak at 260 nm—characteristic of DNA—in IONP@H_1_THs [58]. Dynamic light scattering (Figure 1h) and zeta potential (Figure 1i) measurements showed that, after DNA modification, the hydrodynamic diameter increased from 31 to 46 nm, while the zeta potential shifted from 31.2 ± 0.6 to −6.1 ± 1.3 mV. These changes are consistent with the presence of DNA on the IONP surface. Atomic force microscopy further confirmed successful modification of DNA, revealing numerous low‐height DNA strands surrounding the nanoparticles (Figure 1j). TEM analysis demonstrated that IONP@H_1_THs retained a dispersed state following functional DNA conjugation (Figure 1k).

### 2.2 Responsive Assembly and Self‐Limited Magnetothermal Effect

**FIGURE 2:**
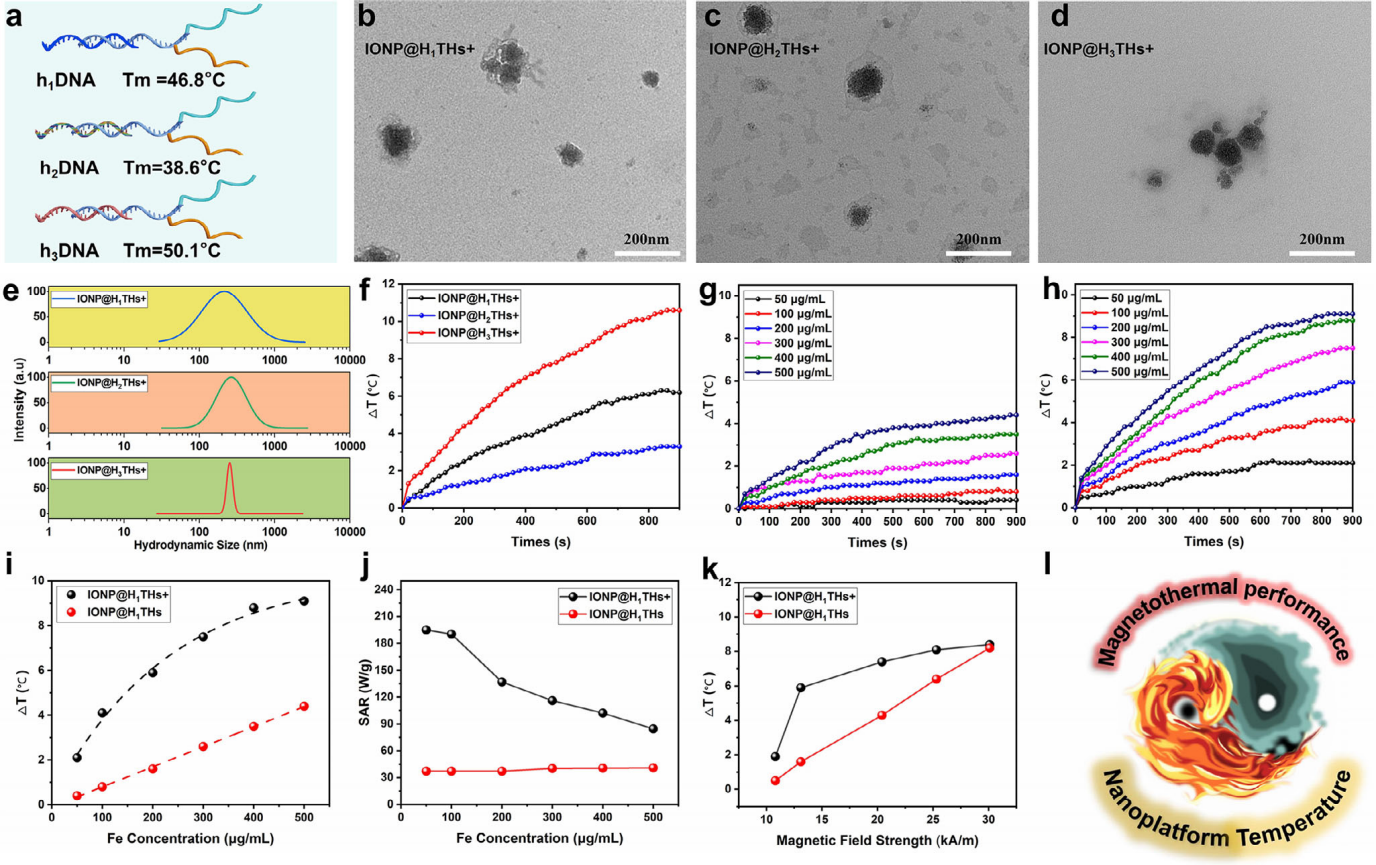
Responsive self‐assembly and magnetothermal properties of different nanoplatforms. (a) Schematic illustration of three hairpin DNAs with varied Tm values used for nanoplatform preparation. (b–d) TEM images of IONP@H_1_THs + Target, IONP@H_2_THs + Target, and IONP@H_3_THs + Target, respectively. (e) Hydrodynamic sizes of the different nanoplatforms after the addition of Target. (f) Temperature–time profiles of the three nanoplatforms at a concentration of 0.2 mg/mL Fe in aqueous solution (initial temperature: 37°C) under AMF (f = 345 kHz, field intensity = 13.1 kA/m). (g, h) Temperature–time profiles of IONP@H_1_THs and IONP@H_1_THs + Target at various Fe concentrations (f = 345 kHz, field intensity = 13.1 kA/m), respectively. (i, j) Temperature elevation and SAR values of IONP@H_1_THs and IONP@H_1_THs + Target at various Fe concentrations (f = 345 kHz, field intensity = 13.1 kA/m), respectively. (k) Temperature elevation of IONP@H_1_THs and IONP@H_1_THs + Target at various magnetic field intensities (f = 345 kHz, field intensity = 10.8, 13.1, 20.4, 25.3, and 30.1 kA/m). (l) Schematic illustration depicting the self‐regulating magnetic heating behavior of the nanoplatform in relation to its temperature.

The hairpin DNA strand plays a crucial role in facilitating nanoparticle aggregation and is strongly influenced by temperature, a key parameter in designing a self‐limiting magnetothermal nanoplatform. In addition to H_1_‐DNA (Tm = 46.8°C), two other hairpin DNA strands with different Tm values (H_2_‐DNA, Tm = 38.6°C; H_3_‐DNA, Tm = 50.1°C) were employed to investigate the relationship between Tm values of hairpin DNA and self‐limiting magnetothermal performance (Figure 2a). As shown in Figure 2b–d, exposure to the target DNA induced pronounced aggregation in all three nanoplatforms, resulting in similar hydrodynamic sizes of approximately 200–300 nm (Figure 2e), whereas random nucleic acids had no effect on nanoparticle dispersion (Figure S2). Heating profiles revealed that the temperature increase was positively correlated with the Tm value of the hairpin DNA (Figure 2f). Among the three, H_1_‐DNA generated a temperature rise of 6.2°C, making it suitable for MTT in cancer therapy while effectively activating Hsp70 [34]. Based on these findings, H_1_‐DNA was selected for further studies.

To compare the magnetic heating performance of assembled IONP@H_1_THs with that of their monodisperse counterparts, we evaluated both forms across varying iron concentrations. As shown in Figure 2g,h, assembled IONP@H_1_THs exhibited superior magnetic heating performance compared with monodisperse IONP@H_1_THs at equivalent concentrations. For monodisperse IONP@H_1_THs, within a concentration range up to 500 µg/mL, a clear linear relationship was observed between temperature rise and nanoparticle concentration. In contrast, aggregated IONP@H_1_THs showed a positive correlation between temperature increase and concentration within the same range; however, the magnitude of the temperature rise declined markedly at higher concentrations, plateauing at approximately 400 µg/mL. Increasing Fe concentration from 400 to 500 µg/mL resulted in only a 0.3°C rise in temperature (Figure 2i). Specific absorption rate (SAR) analysis over 0–900 s revealed that the SAR values for monodisperse IONP@H_1_THs remained relatively stable with increasing Fe concentration. For aggregated IONP@H_1_THs, SAR was substantially higher than that of monodisperse nanoparticles at 50 µg/mL; however, SAR decreased progressively with increasing Fe concentration, eventually approaching that of the monodisperse form (Figure 2j). This behavior may result from elevated temperatures disrupting the DNA hybrids, thereby altering the aggregated state and dynamically modulating magnetic heating performance. To test this hypothesis, both monodisperse and aggregated IONP@H_1_THs were assessed at identical concentrations under varying magnetic field intensities. For monodisperse IONP@H_1_THs, magnetic heating performance increased proportionally with field intensity within 30.1 kA/m. For aggregated IONP@H_1_THs, they also exhibited enhanced heating performance within 20.4 kA/m. However, beyond this field intensity—corresponding to solution temperatures exceeding 45°C—further increases in field intensity only produced minimal additional heating. At 30.1 kA/m, the solution temperatures of monodisperse and assembled IONP@H_1_THs differed by only 0.4°C (Figure 2k). This convergence is likely due to the transition of H‐DNA from double‐stranded to single‐stranded forms as the temperature rises, reducing aggregation and aligning the magnetic heating behavior.

To investigate this self‐limiting heating mechanism, we monitored the aggregation and distribution of the nanoplatform across a range of temperatures. SYBR Green I fluorescent dye (500 µg/mL) was employed to assess the hybridization status of IONP@H_1_THs + Target under varying thermal conditions. As illustrated in Figure S3, the fluorescence intensity progressively diminished with rising temperature, suggesting a decrease in dsDNA within the system. To further corroborate the aggregation and distribution of nanoplatforms at different temperatures, we utilized DLS to determine their hydrodynamic size. As shown in Figure S4a, at an initial temperature of 37°C, the hydrodynamic size of IONP@H_1_THs + Target displayed a near‐Gaussian distribution with an average size of approximately 223 nm. With an increase in temperature after AMF treatment, the hydrodynamic size progressively decreased (Figure S4b–d), and the size distribution exhibited a shift from a near‐Gaussian to a non‐Gaussian pattern, characterized by the presence of two or more distinct size distribution regions. To further explore the mechanism of self‐limiting heating, another concentration of IONP@H_1_THs + Target (400 µg/mL, a concentration that generated similar heating performance with 500 µg/mL from Figure 2h–j) was used here as a comparison to study differences in aggregation and distribution of nanoplatforms. As shown in Figure S4e, after AMF treated 15 min, the hydrodynamic size also followed a non‐Gaussian distribution. Further statistical analysis of the distribution frequency revealed that, in comparison to the 500 µg/mL, the 400 µg/mL nanoplatform exhibited a higher distribution frequency within the size range > 100 nm. Interestingly, the product of distribution frequency and concentration, representing the absolute number of distributed particles, remained consistent. Consequently, there exists a dynamic inverse self‐regulating relationship between the temperature of the nanoplatform and its magnetic heating properties (Figure 2l).

### 2.3 Cellular Uptake and AMF‐Induced Hsp70 Promoter Activation

**FIGURE 3:**
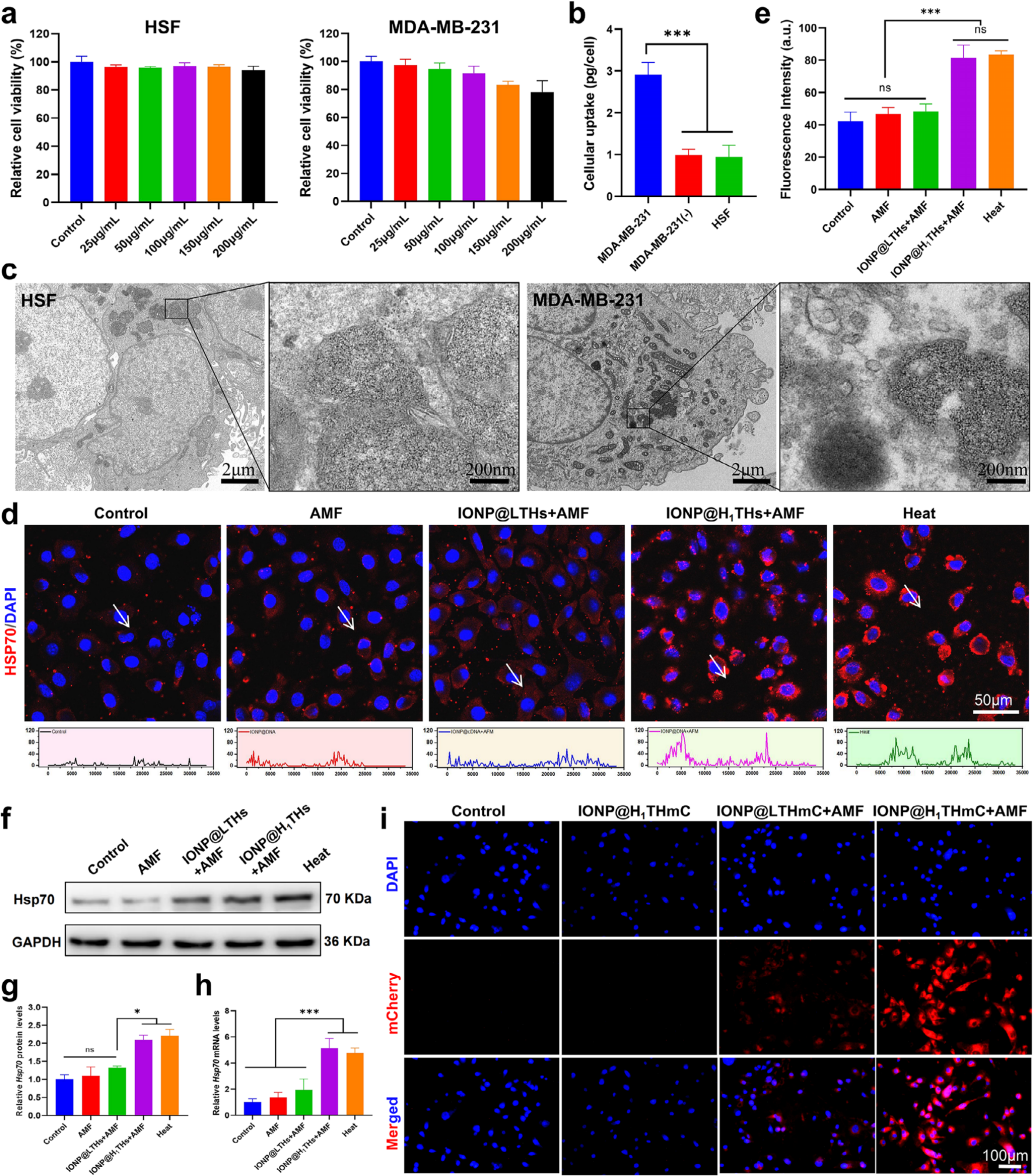
In vitro analysis of cytotoxicity, cellular uptake, and Hsp70 promoter activation. (a) Relative cell viability of HSF and MDA‐MB‐231 cells after different concentrations of IONP@H_1_THs treatment (n = 3). (b) Quantitative analysis of cellular uptake of IONP@H_1_THs (n = 3). MDA‐MB‐231(‐), the cells treated with a nanoplatform without Aptamer DNA. (c) TEM images of HSF and MDA‐MB‐231 cells after IONP@H_1_THs treatment. (d‐e) IFA staining and quantitative analysis of Hsp70 expression in MDA‐MB‐231 cells after different treatments (n = 3). Heat, the cells were directly heated at 43°C for 30 min, respectively. (f‐g) Western blot and quantitative analysis of Hsp70 expression in MDA‐MB‐231 cells after different treatments (n = 3), respectively. (h) Quantitative real‐time polymerase chain reaction analysis of Hsp70 mRNA expression in MDA‐MB‐231 cells after different treatments (n = 3). (i) AMF‐mediated specific activation of Hsp70 promoter using a reporter gene of mCherry (n = 3). Mean ± SD. ^*^ *p*<0.05; ^***^ *p*<0.001; ns, not significant; one‐way ANOVA followed by Tukey post hoc test (b, e, g, h).

The cytotoxicity of IONP@H_1_THs was first assessed in human normal fibroblast (HSF) cells and MDA‐MB‐231 tumor cells using the CCK‐8 assay. As shown in Figure 3a, IONP@H_1_THs exhibited negligible cytotoxic effects on HSF cells. In MDA‐MB‐231 cells, increasing concentrations of IONP@H_1_THs induced only a slight reduction in cell proliferation, with relative viability remaining above 80% after 24 h of exposure to 200 µg/mL. The higher cytotoxicity observed in MDA‐MB‐231 cells compared with HSF cells may be attributed to increased nanoparticle uptake (Figure 3b), potentially mediated by the targeting aptamer on the IONP@H_1_THs surface. To further investigate the intracellular distribution of IONP@H_1_THs, TEM imaging was performed on IONP@H_1_THs pretreated cells. As shown in Figure 3c, pronounced nanoparticle aggregation was observed in MDA‐MB‐231 cells, whereas no obvious aggregation was detected in HSF cells. This differential aggregation is likely due to the elevated expression of TERT in MDA‐MB‐231 cells.

To assess the ability of IONP@H_1_THs to activate heat shock protein under AMF exposure (f = 345 kHz, field intensity = 13.1 kA/m), a nanoplatform (IONP@LTHs) comprising DNA sequences of TR‐DNA and Hs‐DNA, along with a linear DNA incapable of inducing self‐assembly to substitute for the hairpin DNA, was employed as a control to investigate the enhanced magneto‐thermal effect resulting from aggregation. Initially, the temperature of cells treated with AMF was monitored, as illustrated in Figure S5. The IONP@H_1_THs+AMF treatment induced a peak temperature approximately 15 min after the application of AMF, followed by slight fluctuations around this temperature. An Indirect immunofluorescence assay (IFA) targeting Hsp70 was then conducted. As shown in Figure 3d, compared with cells treated with AMF alone, the IONP@H_1_THs + AMF group exhibited markedly stronger red fluorescence, with an intensity comparable to that observed following direct in vitro heating at 43°C. In contrast, the IONP@LTHs + AMF group—which lacks the ability to undergo intracellular aggregation—exhibited only weak fluorescence. It indicates that the combined treatment of IONP@H_1_THs and AMF induced a high expression of Hsp70 protein. Quantitative analysis (Figure 3e) confirmed that the fluorescence intensity in the IONP@H_1_THs + AMF group was significantly higher than that in the IONP@LTHs + AMF and AMF‐only groups, but it was not significantly different from that in the direct heating group. These results indicate that Hsp70 activation is primarily driven by aggregation‐induced AMF heating. To further verify this, Hsp70 protein and mRNA expression levels were examined (Figure 3f–h). Relative to control cells, IONP@H_1_THs + AMF treatment markedly upregulated both protein and mRNA levels of Hsp70, reaching values comparable to those induced by direct heating at 43°C. Although the IONP@LTHs + AMF group exhibited a slight increase, the difference from controls was not statistically significant (*p*< 0.05). Collectively, these findings suggest that intracellular aggregation significantly enhances the magnetic heating efficiency of IONP@H_1_THs, thereby promoting effective Hsp70 activation under AMF.

Next, we employed the coding sequence of mCherry, a red fluorescent protein, as the functional DNA to construct the Hsp70 promoter–driven nanoplatform IONP@H_1_THmC, enabling investigation of AMF‐mediated specific activation of the Hsp70 promoter. As a control, we also prepared a nanoplatform of IONP@LTHmC, which lacks aggregation capability, by substituting the hairpin DNA with linear DNA. As shown in Figure 3i, MDA‐MB‐231 cells treated with IONP@H_1_THmC alone exhibited only negligible red fluorescence. In contrast, co‐treatment with AMF and IONP@H_1_THmC induced a strong red fluorescence signal within the cells. For the IONP@LTHmC + AMF group, only faint red fluorescence was observed, likely due to the minimal heat generation from monodispersed IONP@LTHmC under AMF exposure. These findings indicate that IONP@H_1_THmC combined with AMF robustly activates Hsp70 promoter–driven transcription in cells, an effect attributable to aggregation‐enhanced magnetic heating performance.

### 2.4 In Vitro Anti‐Tumor Effect

**FIGURE 4:**
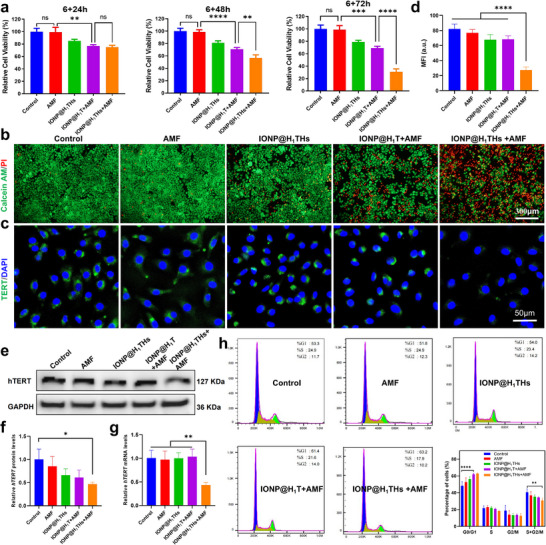
Anti‐tumor effect in vitro. (a) Relative cell viability of MDA‐MB‐231 cells after the treatments with different times (n = 3). (b) Fluorescence images of MDA‐MB‐231 cells after different treatments obtained by Calcein‐AM and PI staining (n = 3). (c‐d) IFA images and quantitative analysis of TERT expression in MDA‐MB‐231 cells after different treatments (n = 3). (e, f) Western blot and quantitative analysis of TERT protein expression in MDA‐MB‐231 cells after different treatments (n = 3). (g) Quantitative analysis of TERT mRNA expression in MDA‐MB‐231 cells after different treatments (n = 3). (h) Cell cycle analysis of MDA‐MB‐231 cells after different treatments (n = 3). Mean ± SD. ^*^ *p*<0.05; ^**^ *p*<0.01; ^***^ *p*<0.001; ^****^ *p*<0.0001; ns, not significant; one‐way ANOVA followed by Tukey post hoc test (a, d, f, g, h).

We first assessed the in vitro anti‐tumor effect of various treatments using the CCK‐8 assay. A nanoplatform consisting of H_1_‐DNA and TR‐DNA (IONP@H_1_T), which lacks the capability for in situ siRNA synthesis upon thermal stimulation, was employed as a control here. MDA‐MB‐231 cells were exposed to IONP@H_1_THs or IONP@H_1_T for 6 h, after which the nanoparticles were removed and replaced with fresh medium. AMF treatment (f = 345 kHz, field intensity = 13.1 kA/m) was then applied for 30 min, followed by incubation for 24, 48, or 72 h before viability assessment. As shown in Figure 4a, at all time points, no statistically significant difference was observed between the control and AMF‐only groups, indicating that AMF alone had no impact on cell proliferation. At 24 h post‐AMF, no significant difference was detected between the IONP@H_1_T + AMF and IONP@H_1_THs + AMF groups; however, both exhibited significantly reduced cell survival compared with the control group (*p*< 0.01), likely due to the cytotoxicity of the nanoparticles combined with their AMF‐induced thermal effects. By 48 h post‐AMF, a significant difference in survival emerged between the IONP@H_1_T + AMF and IONP@H_1_THs + AMF groups (*p*< 0.01), which became more pronounced at 72 h (*p*< 0.0001). Calcein‐AM/PI staining confirmed these trends (Figure 4b). In addition, analysis of reactive oxygen species and apoptosis levels (Figure S6) showed the highest degree in the IONP@H_1_THs + AMF group. These enhanced anti‐tumor effects are likely attributable to decreased TERT expression in IONP@H_1_THs + AMF treatment.

To verify the specific knockdown of TERT following IONP@H_1_THs + AMF treatment, TERT protein level was analyzed by immunofluorescence assay (Figure 4c). Strong fluorescence signals were detected in the control, AMF‐treated, IONP@H_1_THs‐treated, and IONP@H_1_T + AMF‐treated cells. In contrast, cells treated with IONP@H_1_THs + AMF exhibited only faint fluorescence, indicating a marked reduction in TERT expression. A quantitative analysis of the mean fluorescence intensity revealed a statistically significant reduction in the IONP@H_1_THs + AMF group relative to the other four groups (Figure 4d). To further validate this effect, Western blot and quantitative real‐time polymerase chain reaction were performed. As shown in Figure 4e,f, a significant downregulation of TERT protein was observed exclusively in the IONP@H_1_THs + AMF group. Although treatment with IONP@H_1_T + AMF or IONP@H_1_THs alone resulted in slightly lower expression levels compared to controls, these differences were not statistically significant (*p*> 0.05). A similar pattern was detected at the mRNA level (Figure 4g). Collectively, these findings indicate that the enhanced magnetothermal effect generated by nanoparticle aggregation under AMF activates the Hsp70 promoter, triggering siRNA synthesis and leading to targeted suppression of TERT.

Given the pivotal role of TERT in cellular physiological functions, we next examined the effect of IONP@H_1_THs + AMF treatment on cell cycle distribution (Figure 4h). This treatment led to a significant increase in the proportion of cells in the G0/G1 phase, accompanied by a marked decrease in the proportion of cells in the S + G2/M phases. These changes suggest that IONP@H_1_THs + AMF markedly disrupts cell cycle progression, thereby impairing tumor cell proliferation. In contrast, cells treated with IONP@H_1_T + AMF exhibited no significant difference from control cells in the S + G2/M phases, indicating that TERT knockdown synergistically enhances the antitumor efficacy of magnetothermal therapy.

### 2.5 In Vitro Transcriptome Analysis and Verification

**FIGURE 5:**
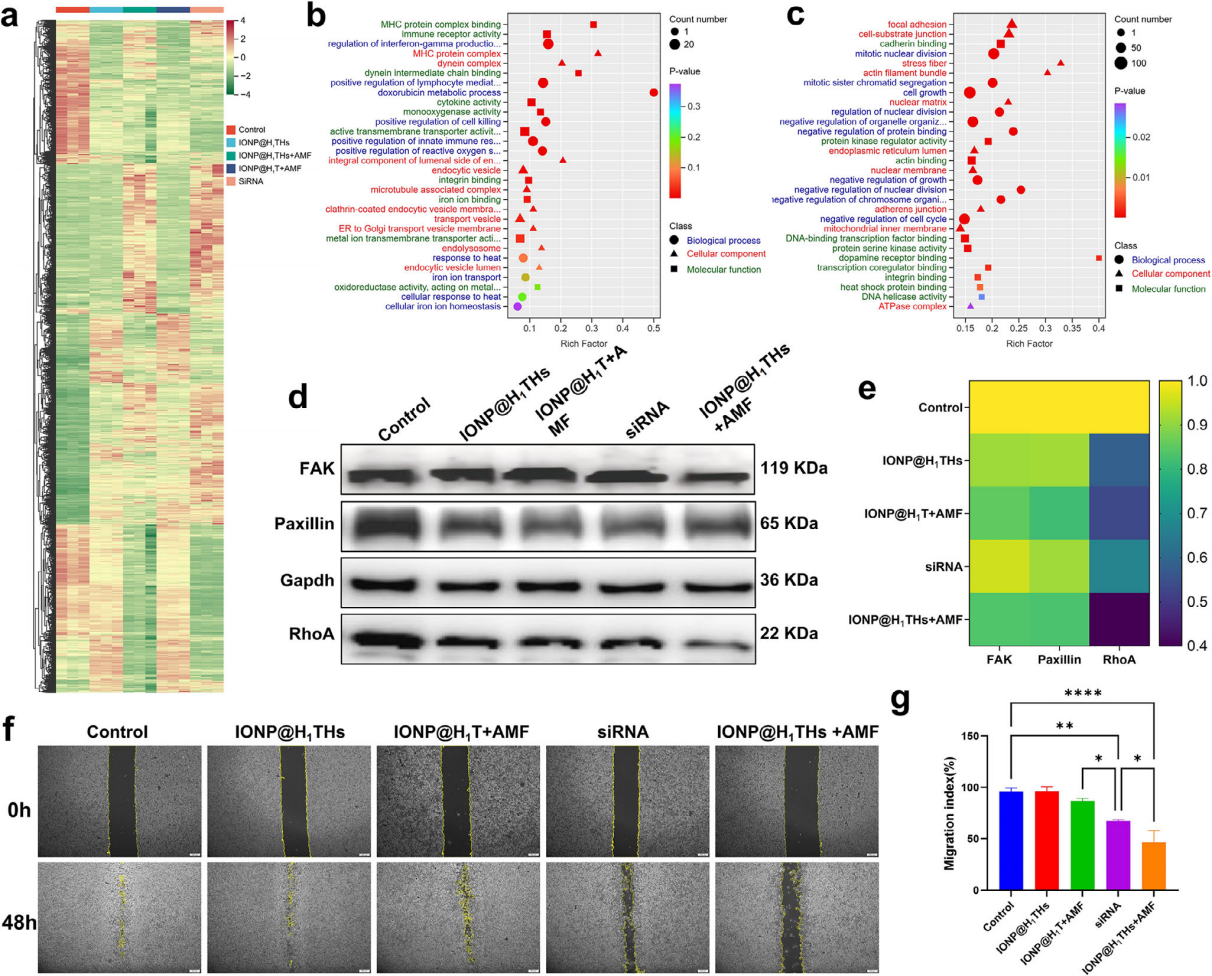
In vitro anti‐tumor mechanism. (a) Heat‐map showing the differentially expressed genes in MDA‐MB‐231 cells after different treatments. (b‐c) GO enrichment analysis of up‐regulated and down‐regulated differentially expressed genes between the control and IONP@H_1_THs treated MDA‐MB‐231 cells, respectively. (d, e) Western blot and quantitative heat‐map of FAK, Paxillin, and RhoA expression in MDA‐MB‐231 cells after different treatments (n = 3). (f, g) Cell migration analysis of MDA‐MB‐231 cells after different treatments (n = 3). Mean ± SD. ^*^ *p*<0.05; ^**^ *p*<0.01; ^****^ *p*<0.0001; one‐way ANOVA followed by Tukey post hoc test (g).

To further elucidate the antitumor mechanism of the mild magnetothermal therapy combined with TERT‐targeted gene knockdown, we performed transcriptome analysis of treated cells. Cells treated with siRNA and IONP@H_1_T + AMF served as controls to assess the synergistic effect of TERT‐knockdown with magnetothermal therapy. As shown in Figure S7, principal component analysis revealed distinct clustering among these groups, and samples within the same group clustered closely, indicating high biological reproducibility. The separation between treatment groups reflected substantial differences in global transcriptional expression, providing a robust foundation for subsequent differential gene screening. A clustering heatmap of differentially expressed genes (DEGs) across all treatments (Figure 5a) showed that, compared with control cells, all four treatments induced marked transcriptional changes involving a large number of DEGs. Venn diagram analysis (Figure S8) revealed 104 common DEGs in four comparisons. Notably, relative to the IONP@H_1_T + AMF vs Control comparison, the IONP@H_1_THs + AMF vs Control comparison showed 3,362 unique DEGs. Further analysis of IONP@H_1_THs + AMF vs IONP@H_1_T + AMF confirmed distinct transcriptional profiles between the two treatments, likely attributable to TERT suppression.

To investigate the potential biological effects of the upregulated and downregulated genes between the IONP@H_1_THs + AMF group and the control group, we performed Gene Ontology (GO) enrichment analysis across three categories: molecular function, biological process, and cellular component. In the enrichment analysis of upregulated genes (Figure 5b), the GO molecular function terms included MHC protein complex binding, immune receptor activity, iron ion binding, and metal ion transmembrane transporter activity, among others. Notably, these functions were predominantly associated with immunity and Fe ion metabolism. Previous studies have demonstrated that upon extensive internalization of IONPs, Fe can undergo Fenton reactions within acidic intracellular compartments, such as lysosomes, generating reactive oxygen species and thereby inducing ferroptosis [59], a phenomenon also observed in our study (Figure S6). In addition, mild hyperthermia can also induce immunogenic cell death and synergize with ferroptosis [3, 60]. In the GO biological process category, several immune‐related terms were also enriched here, including regulation of interferon‐gamma production and positive regulation of lymphocyte‐mediated immunity. Given that these immune effects are already well established in the previous studies, they were not the primary focus of this study. The enrichment of the term “response to heat” further supported the occurrence of AMF‐induced magnetothermal effects at the cellular level. In the GO cellular component category, many terms related to transport vesicles were enriched, primarily reflecting nanoparticle internalization and intracellular trafficking.

In addition, we performed a GO enrichment analysis based on the downregulated genes (Figure 5c). Many of the enriched biological process and molecular function terms were associated with cell division, including mitotic nuclear division, negative regulation of the cell cycle, and DNA helicase activity. These changes are directly linked to the suppression of cell proliferation, and the observed negative regulation of the cell cycle is consistent with our flow cytometry results (Figure 4h). Notably, in the cellular component category, focal adhesion (FA)‐related structures were significantly reduced in IONP@H_1_THs + AMF‐treated cells compared with control cells. FAs are dynamic complexes connecting cells to the extracellular matrix, and they play crucial roles in tumor progression by regulating migration, invasion, proliferation, and survival [61, 62]. Key FA molecules (e.g., FAK and integrins) and their inhibitors (e.g., the FAK inhibitor defactinib) have emerged as promising therapeutic targets and have shown potential in preclinical and clinical studies for suppressing tumor growth [63, 64]. Thus, the observed FA downregulation in our study may represent an additional anti‐tumor mechanism alongside magnetic hyperthermia and ferroptosis. To further investigate FA alterations, we conducted a Gene Set Enrichment Analysis (Figure S9), which confirmed that IONP@H_1_THs + AMF treatment resulted in marked downregulation of FA‐related genes compared with control cells.

To examine FA‐related protein expression under different treatment conditions, we assessed key markers, including FA kinase (FAK), Paxillin, and RhoA, using Western blot analysis. As shown in Figure 5d,e, IONP@H_1_THs + AMF treatment resulted in simultaneous downregulation of FAK, Paxillin, and RhoA, further confirming FA suppression. Tumor cell migration is a pivotal determinant of malignant progression and distant metastasis [65]. Given the central role of FA in regulating cell migration, we next evaluated the impact of IONP@H_1_THs + AMF on migration rates (Figure 4f,g). Neither AMF alone nor IONP@H_1_THs alone significantly affected migration compared with the control group. In contrast, both siRNA and IONP@H_1_THs + AMF treatments markedly reduced migration rates, with IONP@H_1_THs + AMF producing a significantly greater inhibitory effect than siRNA.

### 2.6 Specific Activation of Hsp70 Promoter In Vivo with Self‐Limited Magnetothermal

The precision of in vivo gene editing is a key determinant of the translational potential of GT. Recently, lipid‐based selective organ‐targeted delivery systems have demonstrated considerable potential for gene delivery with high selectivity toward the heart, liver, spleen, lungs, and kidneys. However, these strategies still exhibit a degree of non‐specific delivery and have seldom been applied to tumor targeting [66, 67, 68, 69]. In this study, we aimed to achieve tumor‐specific gene editing via tumor‐responsive aggregation of IONP@H_1_THs, which in turn enhanced the magnetothermal effect of IONP@H_1_THs to specifically activate the gene editing system. To validate this strategy, we employed an mCherry reporter construct driven by the Hsp70 promoter (IONP@H_1_THmC). Activation of the Hsp70 promoter induces downstream mCherry expression, allowing fluorescence‐based visualization of in vivo gene activation. Previous studies have shown that, within a temperature range of 37°C–45°C, Hsp70 promoter activity increases with temperature, whereas excessive heating beyond this range may suppress activity due to tumor cell necrosis [48, 70].

**FIGURE 6:**
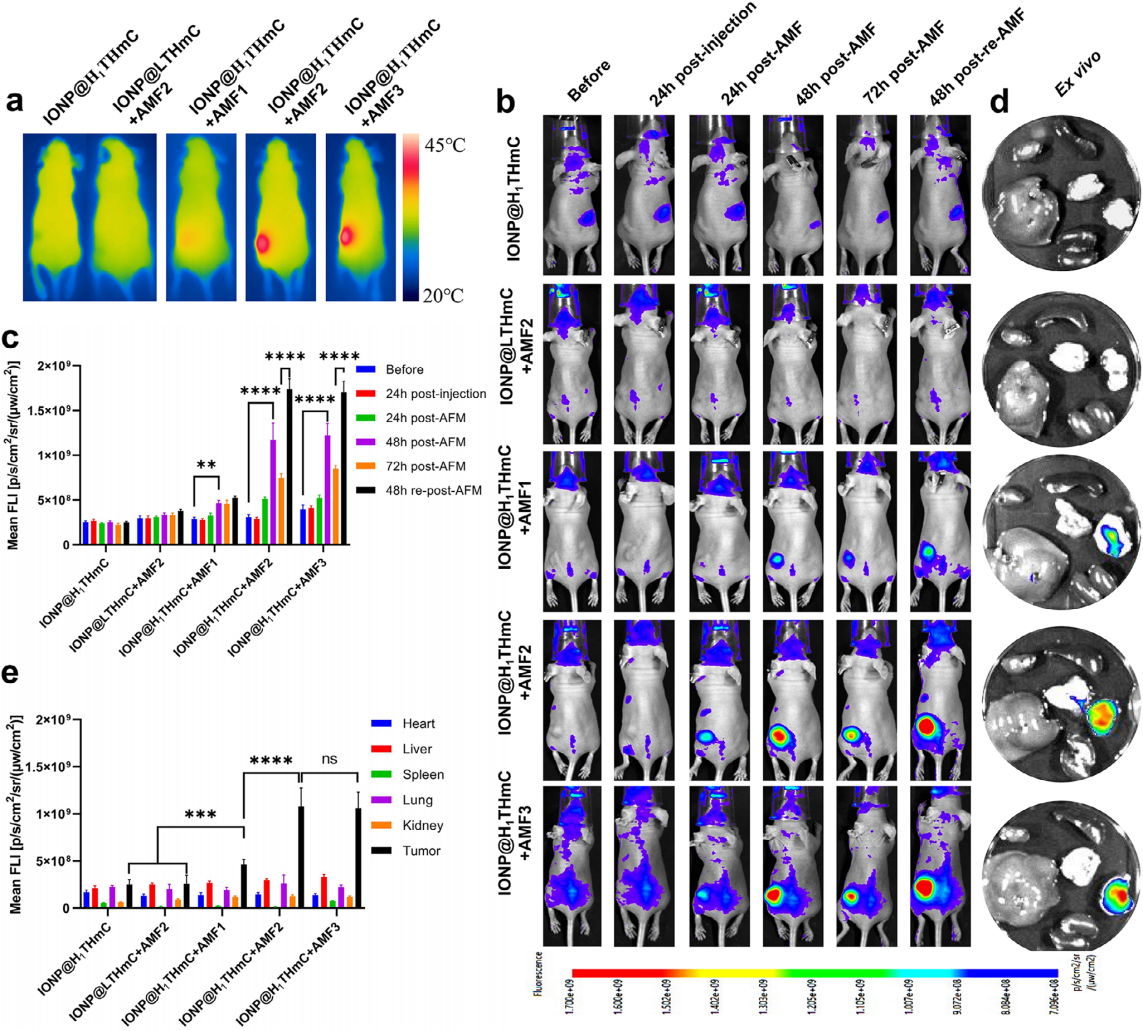
Self‐limited magnetothermal and specific activation of Hsp70 promoter in vivo. (a) Thermal IR images of mice after different treatments. AMF1: 10.8 kA/m; AMF2:13.1 kA/m; AMF3: 20.4 kA/m. (b) In vivo fluorescent images of mice at varied time points after different treatments (n = 3). (c) Fluorescent intensity analysis of tumors in mice at varied time points after different treatments (n = 3). (d, e) Ex vivo fluorescent images and quantitative analysis of tumors and major organs in mice after different treatments (n = 3). Mean ± SD. ^**^ *p*<0.01; ^***^ *p*<0.001; ^****^ *p*<0.0001; one‐way ANOVA followed by Tukey post hoc test (c, e).

To assess the temperature self‐limiting capability of IONP@H_1_THmC, three magnetic field intensities (10.8, 13.1, and 20.4 kA/m) were selected to induce the heating of mice according to the in vitro magnetic field intensity–temperature profile to modulate heating performance. As shown in Figure S10, the control nanoplatform (IONP@LTHmC, a nanoplatform without self‐assembly property) demonstrated limited magnetic heating capabilities under a magnetic field strength of 13.1 kA/m. In contrast, IONP@H_1_THmC exhibited a significant increase in tumor temperature at this magnetic field strength, reaching approximately 42°C, which was apparently higher than that at 10.8 kA/m. However, when the magnetic field strength further increased to 20.4 kA/m, there was no further increase in tumor temperature; instead, it remained consistent with the temperature observed at 13.1 kA/m, indicating a satisfactory self‐limiting temperature property. The terminal temperature of tumors was also detected by infrared imaging and is shown in Figure 6a and Figure S11. The tumor temperature in mice treated with IONP@LTHmC under a 13.1 kA/m field showed no significant difference with control mice (*p*< 0.05), while treatment with IONP@H_1_THmC under a 10.8 kA/m field produced an obvious tumor temperature elevation. Increasing the intensity to 13.1 kA/m further led to a marked temperature rise, whereas further escalation to 20.4 kA/m resulted in only a marginal additional increase of 0.6°C (*p*< 0.05). These findings indicate that the IONP@H_1_THmC nanoplatform exhibits effective temperature heating and self‐regulation properties in vivo.

In vivo fluorescence imaging was performed to evaluate mCherry gene expression in mice non‐invasively (Figure 6b, c). Before AMF treatment, no detectable fluorescence was observed in all groups. At 24 h post‐treatment, weak fluorescence was present in the 10.8 kA/m group, whereas distinct signals were detected in both the 13.1 and 20.4 kA/m groups, with no statistically significant difference between them. By 48 h, fluorescence intensity in the 13.1 and 20.4 kA/m groups increased further. At 72 h, the signal began to decline; however, re‐administration of AMF at this time point led to a marked increase in fluorescence 48 h later. Subsequent ex vivo fluorescence imaging of excised tumors and major organs—including the heart, liver, spleen, lung, and kidney—revealed strong tumor‐localized fluorescence in the 13.1 and 20.4 kA/m groups but with no significant difference (Figure 6d,e).

### 2.7 In Vivo Anti‐Tumor Effect

Prior to initiating therapeutic research trials, we first evaluated the in vivo safety of the IONP@H_1_THs nanoplatform. Both monotherapy with IONP@H_1_THs and its combination with AMF were involved. Blood biochemical analyses were performed on days 1 and 14 following administration of IONP@H_1_THs or IONP@H_1_THs + AMF. No significant changes were observed in serum AST, TP, TBIL, or UREA levels compared with the normal control group (Figure S12). Furthermore, histological examination of major organs by H&E staining revealed no apparent pathological alterations under either treatment condition at either time point (Figure S13).

**FIGURE 7:**
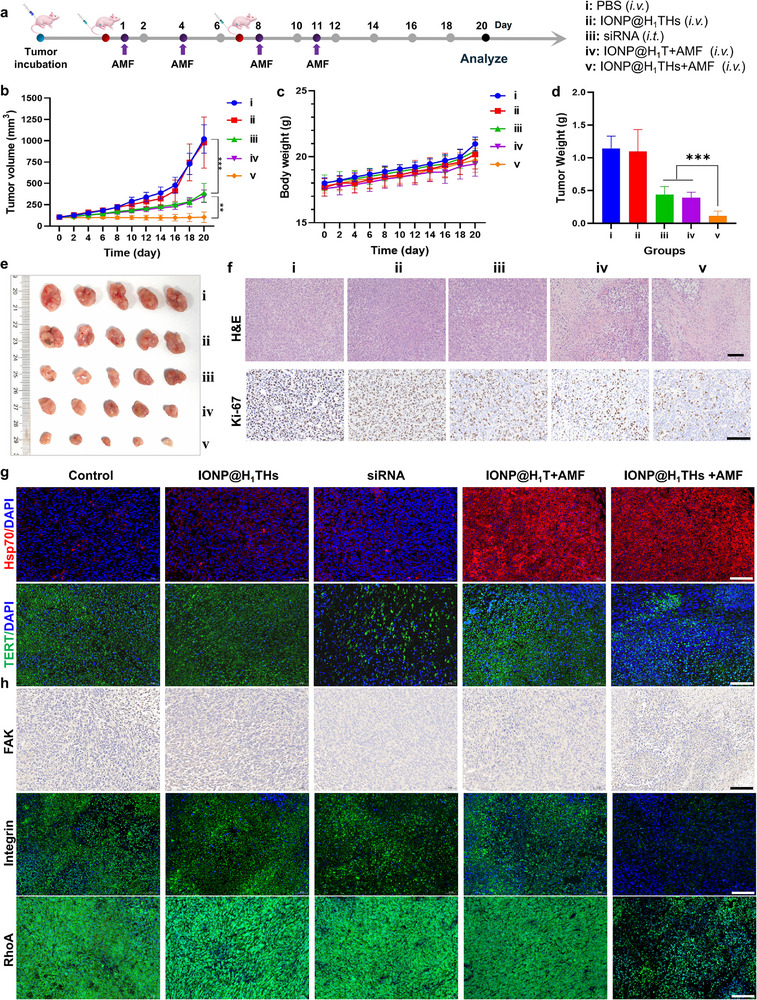
Anti‐tumor effect of IONP@H_1_THs + AMF in vivo. (a) Schematic diagram of treatment schedule. (b) Tumor volume growth curves in vivo (n = 5). (c) The body weight change within the observation period (n = 5). (d‐e) The ex vivo images and weight of tumors on day 20 (n = 5), respectively. (f) Images of H&E and Ki‐67 staining of tumor tissues after different treatments (n = 3). (g) Immunofluorescence images of Hsp70 and TERT in tumor tissues after different treatments (n = 3). (h) Immunofluorescence or immunohistochemical images of FAK, Integrin, and RhoA in tumor tissues after different treatments (n = 3). bar:100 µm. Mean ± SD.^**^ *p*<0.01;^***^ *p*<0.001; student's t‐test in (b), while one‐way ANOVA followed by Tukey post hoc test in (d).

The in vivo antitumor efficacy of various treatments—including PBS, IONP@H_1_THs, siRNA, IONP@H_1_T + AMF, and IONP@H_1_THs + AMF—was evaluated in an MDA‐MB‐231 unilateral tumor‐bearing mouse model, following the scheme outlined in Figure 7a. When tumor volumes reached approximately 100 mm^3^, drug administration was initiated, followed by AMF exposure (f = 345 kHz, field intensity = 13.1 kA/m) at 24 and 72 h post‐administration to induce localized tumor heating. As shown in Figure 7b, tumor volumes in the PBS and IONP@H_1_THs groups increased rapidly over time. Both the IONP@H_1_T + AMF and siRNA treatment groups exhibited significant tumor inhibition when compared with the PBS group. And as expected, the IONP@H_1_THs + AMF treatment demonstrated the most anti‐tumor effect, also with significantly smaller tumor volumes compared to those observed in the IONP@H_1_T + AMF (*P*<0.01) or siRNA (*P*<0.001) treatment groups. This enhanced efficacy is likely due to the synergistic action of magnetothermal therapy and TERT suppression. Body weights were monitored throughout the study, with only a slight increase observed in the PBS and IONP@H_1_TH groups during the later stages, likely reflecting the rapid growth of tumors (Figure 7c). Tumor weight (Figure 7d) and ex vivo tumor image (Figure 7e) showed trends consistent with the tumor volume data.

To further investigate the histological alterations in tumor tissues under different treatment regimens, H&E staining and Ki‐67 expression analysis were first performed (Figure 7f). The results showed that both IONP@H_1_T + AMF and IONP@H_1_THs + AMF treatments markedly induced tumor cell death (Figure S14). Ki‐67 immunohistochemical analysis revealed that IONP@H_1_THs monotherapy had no significant effect on Ki‐67 expression (*p*> 0.05), whereas IONP@H_1_T + AMF or siRNA treatment substantially suppressed Ki‐67 expression. Notably, the positive rate of Ki‐67 was more suppressed after IONP@H_1_THs + AMF treatment (Figure S15). Additionally, the TUNEL staining analysis also confirmed that the IONP@H_1_THs + AMF treatment induced the most apoptosis compared with the other four treatments (Figure S16). Subsequently, IFA was conducted to assess Hsp70 expression in tumor tissues. Strong red fluorescence signals were observed in the IONP@H_1_T + AMF and IONP@H_1_THs + AMF groups, indicating that the nanoplatform exhibits robust tumor hyperthermic performance upon AMF exposure (Figure 7g). Quantitative analysis indicated that there was no statistically significant difference in Hsp70 fluorescence intensity between the IONP@H_1_T + AMF and IONP@H_1_THS + AMF treatment groups. However, both treatment groups exhibited significantly higher fluorescence intensity compared to the control group (Figure S17). Furthermore, TERT expression in tumor tissues was also evaluated (Figure 7g). As anticipated, both IONP@H_1_THs + AMF and siRNA treatments reduced TERT expression, supporting the feasibility of the Hsp70 promoter‐mediated in situ siRNA synthesis strategy for gene silencing. The quantitative analysis showed no statistical difference in TERT expression levels between the siRNA group and the IONP@H_1_THs + AMF group, despite the former being slightly lower level (Figure S18). However, the IONP@H_1_THs + AMF group exhibited a more significant therapeutic effect after combined magnetothermia.

Finally, to elucidate the anti‐tumor mechanism of IONP@H_1_THs + AMF, we examined the expression of FAK, integrin, and RhoA in tumor tissues—key components of the FAK signaling pathway. IONP@H_1_THs + AMF treatment concurrently downregulated integrin, FAK, and α‐actin expression (Figure 7h and Figures S19–S21), consistent with the trends observed in vitro. Given the pivotal role of the FA signaling pathway in tumor initiation, progression, and metastasis, its suppression is expected to reduce pathway activity, thereby inhibiting malignant tumor progression.

In recent years, the integration of hyperthermia and GT has demonstrated considerable promise in the field of cancer therapeutics [71, 72]. When selecting a hyperthermia platform, notably, beyond the magnetic heating platform utilized in the present study, several alternative modalities exist, including near‐infrared lasers and focused ultrasound [72, 73]. Nevertheless, near‐infrared laser‐based hyperthermia is hampered by suboptimal tissue penetration depth and inherent risks of optical path‐related toxicity in deep‐seated tissues [74]. Although focused ultrasound offers superior tissue penetration capabilities, it is prone to inducing cavitation effects within bodily fluids—a phenomenon that may compromise the integrity of blood vessels or cellular structures, thereby potentially triggering hemorrhage or inflammatory responses [75]. Furthermore, focused ultrasound‐mediated hyperthermia typically necessitates image guidance, such as magnetic resonance imaging and real‐time ultrasound, to ensure targeting accuracy [76]. In the field of GT combined with hyperthermia treatment, the two most prevalent strategies are heat‐induced in situ synthesis and heat‐induced release of therapeutic genes. Compared with heat‐induced release, in situ synthesis exhibits enhanced specificity; importantly, the incorporation of heat‐responsive elements further augments the targeting precision of GT.

In the current study, a targeted response system was developed to recognize the overexpression of TERT mRNA in cancer cells. This system induces the aggregation of ultra‐small magnetic nanoparticles and subsequent enhancement of their magnetic heating performance, which in turn enables the specific induction of hyperthermia in cancer cells and facilitates the in situ synthesis of therapeutic genes, thus conferring remarkable specificity to GT applications. Additionally, to mitigate potential adverse effects arising from excessive hyperthermia during the optimization of magnetic heating efficiency, an innovative reversible dynamic assembly strategy based on DNA was introduced, which enables temperature‐limited magnetic hyperthermia, a critical feature for improving therapeutic safety. In comparison with existing temperature‐limiting approaches—such as deferiprone‐induced charge transfer blockade methods—the DNA hybridization‐based temperature‐sensitive strategy offers distinct advantages, including reversible responsiveness and more precise temperature regulation.

## 3 Conclusion

In conclusion, we designed and successfully fabricated a novel nanoplatform (IONP@H_1_THs) composed of ultra‐small IONP and functional DNAs for precision synergistic cancer therapy via mild‐thermal therapy and heat‐induced TERT silencing. In a monodisperse state, IONP@H_1_THs exhibited limited magnetic heating capacity and low cytotoxicity. Upon interaction with TERT mRNA, hairpin DNA‐mediated self‐assembly induced spontaneous aggregation and then enhanced the magnetothermal efficiency. Under an AMF, heating to a defined threshold temperature triggered the thermosensitive transition of assembled double‐stranded DNA into single strands, thereby reducing aggregation and attenuating magnetic induction heating. This thermo‐self‐limiting effect enabled precise mild‐thermal therapy. Furthermore, the mild heating activated Hsp70 promoter‐driven transcription, leading to in situ siRNA synthesis and effective TERT gene knockdown. Collectively, this work presents a promising approach for precision cancer therapy by integrating mild thermal treatment with heat‐triggered gene silencing.

## 4 Experimental Section

### 4.1 Preparation of IONP@H_1_THs and Controls

Oleic acid‐modified ultra‐small iron oxide nanoparticle (IONP‐OA) was purchased from XFNANO Materials Tech Co., Ltd (Nanjing, China). First, the hydrophobic ligand, oleic acid, on the surface of IONP‐OA was replaced with hydrophilic dopamine molecules using a ligand‐exchange method, with minor modifications based on a previous report [55]. Specifically, 5 mg dried IONP‐OA was dispersed in 0.5 mL chloroform (10 mg mL^−^^1^) using an ultrasonic bath for 5 min. 10 mg dopamine was dissolved in 0.3 mL degassed H_2_O and then added to the IONP‐OA suspension. The mixture was sonicated for 30 min to complete the ligand exchange. The hydrophilic nanoparticle (IONP@DA) was obtained by centrifugation to eliminate free dopamine. The prepared IONP@DA was characterized by transmission electron microscopy (TEM, Hitachi HT‐7700, Hitachi, Tokyo, Japan), High Resolution Transmission Electron Microscopy (HRTEM, FEI Tecnai G2 F30, Hillsboro, USA), X‐ray diffraction (XRD, PANalytical X'Pert Powder, Almelo, Netherlands), and X‐ray photoelectron spectroscopy (XPS, ESCALAB 250Xi, Waltham, MA, USA).

Subsequently, the hybridized DNA contains three functional DNA strands: COOH modified‐hairpin DNA (H‐DNA), targeting‐responsive DNA (TR‐DNA), and Hsp70 promoter‐driven shDNA synthesis strand (Hs‐DNA)—were immobilized on the surface of IONP@DA using EDC/NHS. Hsp70 promoter and its downstream nucleotides were designed with palindrome sequences to simulate double‐stranded DNA. The three DNAs were initially annealed at a molar ratio of 1:1:1. After annealing, the hybridized DNAs were obtained by recovering the complexes from the agarose gel using a TIANgel Purification Kit (TIANgen, Beijing, China). Then they were mixed with IONP@DA in EDC/NHS at a molar ratio of 100:1 at 37°C for 12 h under continuous vibration. Following the reaction, the resultant product was centrifuged, and the precipitate was collected and resuspended in DNase‐free water. The final construct was named IONP@H_1_THs and stored at 4°C for subsequent experiments.

To regulate the melting temperature (Tm) of the hybridized DNA, the length of the complementary region in the hairpin DNA was adjusted. Three variants of hairpin DNA with different Tm values were designed (IDT OligoAnalyzer): H_1_‐DNA (Tm = 46.8°C), H_2_‐DNA (Tm = 38.6°C), and H_3_‐DNA (Tm = 50.1°C). Correspondingly, three nanoplatforms were developed and designated as IONP@H_1_THs, IONP@H_2_THs, and IONP@H_3_THs. Other controls were used, including: IONP@H_1_THmC, the Hs‐DNA was replaced with Hsp70 promoter controlled mCherry fluorescent protein‐encoding gene; IONP@H_1_T comprises DNA sequences including H_1_‐DNA and TR‐DNA, but lacks the functional DNA segment associated with the Hsp70 promoter; IONP@LTHs consists of DNA sequences, including TR‐DNA and Hs‐DNA, whereas the hairpin DNA has been substituted with a linear DNA that was incapable of inducing self‐assembly; IONP@LTHmC, a linear DNA replaced the hairpin DNA, and the Hs‐DNA was replaced with Hsp70 promoter controlled mCherry fluorescent protein‐encoding gene. Detailed DNA sequences are presented in Table S1.

### 4.2 Responsive Aggregation and Magnetothermal Properties of IONP@H_1_THs

The target TERT DNA (the same sequence with TERT mRNA) was added to the solution of IONP@H_1_THs with a molar ratio of 10:1. After 6 h reaction at 37°C, the Nicomp ZLS Z3000 (PSS, Port Richey, FL, USA) was used to detect the hydrodynamic size and Zeta potential, and TEM was also used to directly observe the aggregation. Then preparation of dispersed nanoparticles and aggregated nanoparticles with varying iron (Fe) concentrations (50, 100, 200, 300, 400, and 500 µg/mL), and the magnetic induction heating properties under an alternating magnetic field (AMF) (f = 345 kHz, Field intensity = 13.1 kA/m) were tested using a Super‐Mag M5 induction heating system (Shaanxi Baici Kangda Medical Technology Co., Ltd., China). In addition, the dispersed IONP@H_1_THs and aggregated IONP@H_1_THs with the same Fe concentration (200 µg/mL) were used to test the magnetic induction heating properties under different alternating magnetic fields (f = 345 kHz, Field intensity = 10.8, 13.1, 20.4 and 30.1 kA/m).

### 4.3 Mechanism of Self‐Limited Magnetothermal

IONP@H_1_THs at a concentration of 500 µg/mL Fe were dispersed in 20 µL of PBS containing SYBR Green I within a 200 µL PCR tube. The fluorescence intensity of the mixture was monitored across a temperature range of 37°C to 80°C using an ABI 7500 PCR system (Applied Biosystems). The temperature of IONP@H_1_THs, with Fe concentrations of 400 µg/mL and 500 µg/mL, was measured after AMF treatment using a fiber optic temperature sensor. Specific temperatures at 5, 10, and 15 min post‐treatment were recorded. Subsequently, IONP@H_1_THs with Fe concentrations of 400 and 500 µg/mL were heated and maintained at these specific temperatures, after which the hydrodynamic size was determined using the Nicomp ZLS Z3000.

### 4.4 Cell Culture

MDA‐MB‐231 and HSF cells were purchased from Procell Life Science & Technology Co., Ltd (Wuhan, China). MDA‐MB‐231 cells were cultured in DMEM containing 10% fetal bovine serum and 1% streptomycin/penicillin, and HSF cells were cultured in DMEM supplied with 10% fetal bovine serum, 1% streptomycin/penicillin, 1% Glutamax‐1, 5 ng/ml bFGF, 5 µg/ml recombinant human insulin, 1 µg/ml hydrocortisone, and 50 µg/ml ascorbic acid. Both the cells were cultured at 37°C in a 5% CO_2_ incubator (Thermo, Waltham, MA, USA). Upon the cells reaching the logarithmic growth phase, they were harvested using a trypsin‐EDTA solution (0.25%) and subcultivated for subsequent experiments.

### 4.5 Cellular Uptake

Approximately 5×10^4^ MDA‐MB‐231 or HSF cells were seeded in 6‐well plates to culture overnight, then the media containing IONP@H_1_THs (Fe = 200 µg/mL) was used to replace the original culture media. After 6 h co‐incubation, the IONP@H_1_THs in the extracellular space were removed, and the cells were harvested. Half of the cells were counted and digested with chloroazotic acid (37% hydrochloric acid: 65% nitric acid = 3:1 v/v) for detecting the Fe element quantitatively, the other half were fixed with glutaraldehyde and used for TEM (Hitachi HT‐7800) to observe the distribution of IONP@H_1_THs within the cells.

### 4.6 Cytotoxicity, Cell Cycle, and ROS Detection

The cytotoxicity of IONP@H_1_THs on MDA‐MB‐231 and HSF cells was evaluated using the CCK‐8 assay. Approximately 5 ×10^3^ cells per well were seeded in 96‐well plates to culture overnight, and then treated with different Fe concentrations (0, 25, 50, 100, 150, 200 µg/mL) for 24 h. Then cells were washed with PBS three times and further cultured with a mixed medium containing 10% WST‐8 solution (Beyotime, China) for another 1.5 h. The optical density value was measured at 450 nm using a varioskan flash microplate spectrophotometer (Thermo, Waltham, MA, USA). The relative cell viability was acquired by calculating the percentage of viable cells to control cells.

Five groups, namely PBS, AMF, IONP@H_1_THs, IONP@H_1_T + AMF, and IONP@H_1_THs + AMF, were employed to investigate the killing efficacy on tumor cells. Subsequent to a 6‐h treatment with IONP@H_1_THs or IONP@H_1_T (Fe = 100 µg/mL), the extracellular IONPs were removed, and fresh medium was replenished. Subsequently, AMF treatment (frequency = 345 kHz, field intensity = 13.1 kA/m) was carried out for 30 min. Thereafter, the cells were cultured continuously for an additional 24, 48, and 72 h. At each time‐point, the killing efficacy was detected via the CCK‐8 assay, following a procedure analogous to that of the cytotoxicity assay. Besides CCK‐8 assay, Calcein‐AM and propidium iodide (PI) (Beyotime, China) staining was also used to visualize the cytotoxicity under a standard protocol. Also, for the cells treated for 6+72 h, the cell cycle was analyzed using flow cytometry. ROS generation was determined using a ROS Assay Kit (Beyotime, China). The apoptosis was also detected using a Tunel staining Kit (Beyotime, China). All the fluorescence images were captured using an inverted fluorescence microscope (Leica Microsystems, Wetzlar, Germany).

### 4.7 Magnetothermal‐Mediated Hsp70 Overexpression In Vitro

A nanoplatform (IONP@LTHs), incorporating TR‐DNA, Hs‐DNA, and a linear DNA incapable of inducing self‐assembly as a substitute for hairpin DNA, was employed as a control to investigate the enhanced magneto‐thermal effect due to aggregation. MDA‐MB‐231 cells were seeded into 6‐well plates and cultured overnight to facilitate cell adherence. Subsequently, five distinct treatments were administered: control, AMF only, IONP@LTHs+AMF, IONP@H_1_THs+AMF, and direct heating (43°C for 30 min). Following a 6‐h treatment with IONP@LTHs or IONP@H_1_THs (Fe concentration = 200 µg/mL), AMF (frequency = 345 kHz, field intensity = 13.1 kA/m) was applied for 30 min. After an additional 24‐h culture period, cells were harvested for protein and RNA extraction. The relative expression levels of Hsp70 protein and mRNA were determined using western blotting and quantitative real‐time polymerase chain reaction, respectively. To monitor the temperature in AMF‐treated groups, additional cells were similarly treated with IONP@LTHs or IONP@H_1_THs (Fe concentration = 200 µg/mL) for 6 h. Subsequently, the cells were suspended in 50 µL of PBS within a 200 µL tube, and a fiber optic temperature sensor was utilized to measure the temperature under AMF exposure.

### 4.8 Magnetothermal‐Mediated Hsp70 Promoter Activation In Vitro

The activation of the Hsp70 promoter by magnetothermal was investigated using two control nanoplatforms, IONP@H_1_THmC and IONP@LTHmC. IONP@H_1_THmC was composed of a DNA fragment encompassing the Hsp70 promoter‐regulated mCherry gene, H_1_‐DNA, and TR‐DNA. In contrast, IONP@LTHmC consists of a DNA fragment containing the Hsp70 promoter‐controlled mCherry gene, a linear DNA (lacking the capacity of assembly), and TR‐DNA. This experiment incorporated four groups, specifically the blank control group, the IONP@H_1_THmC group, the IONP@LTHmC + AMF group, and the IONP@H_1_THmC + AMF group. MDA‐MB‐231 cells were seeded in a 24‐well plate and cultured overnight. Subsequently, IONP@H_1_THmC or IONP@LTHmC (Fe = 200 µg/mL) was added to the culture medium and co‐incubated with the cells for 6 h. Following this, the cells were washed thrice with PBS, and fresh culture medium was added. Then the cells were subjected to AMF treatment (frequency = 345 kHz, field intensity = 13.1 kA/m) for 30 min. Subsequently, the cells were cultured continuously for an additional 48 h. Finally, the cells were stained with Hoechst 33342 and imaged using an inverted fluorescence microscope (Leica Microsystems, Wetzlar, Germany).

### 4.9 Western Blotting Assay

The total protein extraction kit (Solarbio, Beijing) and BCA protein quantification kit (Solarbio, Beijing) were employed to extract proteins from the treated cells and quantify the extracted proteins. Subsequently, approximately 20 µg of total proteins from each sample were utilized for sodium dodecyl sulfate polyacrylamide gel electrophoresis, followed by electrophoretic transfer onto a polyvinylidene difluoride membrane. The membrane was blocked with 5% albumin and co‐incubated with anti‐Hsp70 (Solarbio, Beijing), anti‐TERT (Beyotime, China), anti‐RhoA (Beyotime, China), anti‐Paxillin (Beyotime, China), anti‐FAK (Beyotime, China), and anti‐GAPDH (Servicebio, China) antibody at 4°C overnight for specific binding. HRP‐conjugated goat anti‐rabbit IgG antibody was used as the secondary antibody. Finally, after staining with an Enhanced Chemiluminescent Kit (Beyotime, China), the bands were imaged using Image Quant LAS 4000 (GE Healthcare Life Sciences).

### 4.10 Quantitative Real‐Time Polymerase Chain Reaction

The total RNAs were extracted utilizing an RNAsimple Total RNA Kit (Tiangen, China). Subsequently, the mRNAs were transcribed into complementary deoxyribonucleic acid employing a PrimeScript first Strand cDNA Synthesis Kit (Takara, China). Quantitative real‐time polymerase chain reaction was then conducted using TB Green Premix DimerEraser (Takara, China) in an ABI 7500 PCR system (Applied Biosystems). The amplification program was configured as follows: initial denaturation at 95°C for 30 s, followed by 40 cycles consisting of 8 s at 95°C and 35 s at 60°C. The data were normalized to the mRNA levels of the housekeeping gene glyceraldehyde‐3‐phosphate dehydrogenase (GAPDH). The primers of TERT, Hsp70, and GAPDH were purchased from Beyotime (Shanghai, China).

### 4.11 Indirect Immunofluorescence Assay

Following surgical dissection, the tumors were promptly fixed with paraformaldehyde and subsequently embedded in paraffin for tissue sectioning. Paraffin‐embedded tumor sections or directly fixed cultured cells were treated with 1.5% Triton X‐100 for 30 min, followed by incubation with 5% bovine serum albumin for 2 h at room temperature. Subsequently, the cells or slices were incubated overnight at 4°C with antibodies against Hsp70 (Solarbio, China), TERT (Santa Cruz, USA), RhoA (Beyotime, China), Integrin (Beyotime, China), or FAK (Beyotime, China). This was followed by staining with 488‐ or 594‐ labeled goat anti‐rabbit IgG antibody, and DAPI for nuclear staining. The stained cells or tissue sections were examined using an inverted fluorescence microscope (Leica Microsystems, Wetzlar, Germany) or a laser scanning confocal microscope (ZEISS LSM 880, Germany).

### 4.12 Cell Migration Experiment

Five groups were employed to investigate the impact of IONP@H_1_THs + AMF on cell migration, including the control, IONP@H_1_THs, IONP@H_1_T + AMF, siRNA, and IONP@H_1_THs + AMF. MDA‐MB‐231 cells were seeded into a 6‐well plate at a density such that they would achieve 80%–90% confluency following 24–48 h of incubation. Subsequently, a sterile 200 µL pipette tip was utilized to create a straight and uniform scratch across the center of each well by firmly dragging the tip along the well bottom. The wells were then washed three times with PBS to remove floating cells and imaged using an inverted microscope. Next, the cells were subjected to different treatments for 6 h (Fe = 200 µg/mL). After treatment, the cells were washed, and the medium was replaced with fresh medium, and then treated with AMF (f = 345 kHz, Field intensity = 13.1 kA/m) and cultured for an additional 48 h. Finally, the cells were imaged again using an inverted fluorescence microscope (Leica Microsystems, Wetzlar, Germany), and the cell migration indices were calculated based on the changes in areas.

### 4.13 Transcriptome Sequencing

To study the effect of IONP@H_1_THs + AMF on cell transcription, approximately 1×10^5^ MDA‐MB‐231 cells were seeded into 6‐well plates overnight at 37°C. Then, the cells were treated with siRNA, IONP@H_1_THs, IONP@H_1_T + AMF, or IONP@H_1_THs + AMF for 6 h (Fe = 200 µg/mL), and the untreated cells were used as blank control. After treatment, the medium supernatant was removed, and the cells were washed 3 times and replaced with fresh medium, and subjected to AMF (frequency = 345 kHz, field intensity = 13.1 kA/m) for 30 min. Then the cells were cultured for another 48 h. Finally, the cells were washed with cold PBS, and 1 mL of TRIzol reagent was added to lyse the cells for further *RNA‐seq* sequencing (MetWare, Wuhan, China). The data analysis of the transcriptome was performed using the Metware Cloud (https://cloud.metware.cn).

### 4.14 Tumor Model

All animal experiments were carried out in accordance with the guidelines of the National Institutes of Health regarding the ethical utilization of animals in research and were approved by the Laboratory Animal Welfare and Ethics Committee of the Army Medical University (AMUWEC20234941). Mice were housed in a 12 h light/12 h dark cycle with a temperature of 22°C–25°C, and a humidity of 50%. To establish MDA‐MB‐231 tumor xenograft‐bearing models, six‐week‐old healthy female BALB/c nude mice (Tengxin, Chongqing, China) were subcutaneously injected with 1×10^7^ cells per mouse in the dorsal region with Matrigel. The tumor volume was measured using the formula: volume = length × width^2^/2. When the tumor volume reached approximately 80–120 mm^3^, the mice were randomly assigned to different groups for subsequent experiments.

### 4.15 Magnetothermal‐Mediated Hsp70 Promoter Activation In Vivo

Five tumor groups were employed in this study, namely IONP@H_1_THmC, IONP@H_1_THmC + AMF1 (frequency = 345 kHz, field intensity = 10.8 kA/m), IONP@H_1_THmC + AMF2 (frequency = 345 kHz, field intensity = 13.1 kA/m), IONP@H_1_THmC + AMF3 (frequency = 345 kHz, field intensity = 20.4 kA/m), and IONP@LTHmC + AMF2 (frequency = 345 kHz, field intensity = 13.1 kA/m). 24 h after intravenous administration of IONP@H_1_THmC or PBS, the mice were exposed to AMF with corresponding parameters for 30 min and subsequently maintained under standard conditions. The temperature in tumors was monitored by implanting a fiber optic temperature sensor into the tumor. In vivo mouse fluorescence was obtained using an AniView100 system (BLT, Guangzhou, China) prior to H_1_THmC treatment, 24 h after IONP@H_1_THmC treatment, 24 h after AMF, 48 h after AMF, 72 h after AMF, and 48 h after AMF re‐treatment.

### 4.16 In Vivo Anti‐Tumor Effect

To investigate the anti‐tumor efficacy of IONP@H_1_THs + AMF, five groups of MDA‐MB‐231 tumor xenograft‐bearing mice were employed, namely the PBS, IONP@H_1_THs, IONP@H_1_T + AMF, siRNA, and IONP@H_1_THs + AMF. When the tumor volume approximated 80‐120 mm^3^, IONP@H_1_T, IONP@H_1_THs, or PBS was intravenously injected into the mice (Fe = 50 mg/kg body weight). 24 h after injection, the mice in the AMF, IONP@H_1_T + AMF, and IONP@H_1_THs + AMF groups were subjected to AMF treatment (frequency = 345 kHz, field intensity = 13.1 kA/m). Three days subsequent to the first AMF treatment, a second AMF treatment was carried out. After 7 days post‐injection, a second round of treatment was involved. The tumor volume was monitored every two days until 20 days after the initial administration.

### 4.17 In Vivo Toxicity Study

Healthy 6‐week‐old female Balb/c mice (Tengxin, Chongqing, China) were randomly assigned to five groups. The control group mice were intravenously injected with PBS, while the other groups were intravenously injected with IONP@H_1_THs (n = 8, Fe = 50 mg/kg body weight). Blood samples and major organs (heart, liver, spleen, lungs, and kidneys) were collected from mice that were humanely euthanized at 1 and 14 days post‐injection. Regarding blood samples, whole blood was collected from the mice and centrifuged at 2000 g for 10 min to isolate the serum. Biochemical parameters, such as alanine aminotransferase (ALT), total protein (TP), blood urea nitrogen (BUN), and total bilirubin (TBIL), were analyzed using an automated chemistry analyzer following standard protocols. For tissue samples, the tissues were fixed in 4% paraformaldehyde, subsequently embedded in paraffin, sectioned, and subjected to hematoxylin and eosin (H&E) staining. The stained sections were then observed under a bright‐field microscope.

### 4.18 Statistical Analysis

All data were presented as mean values ± standard deviation (SD). The differences between the two groups were analyzed via a one‐tailed Student's t‐test, while the differences among three or more groups were analyzed using ordinary one‐way ANOVA followed by Tukey's post‐hoc test. Comparisons were carried out with GraphPad Prism (San Diego, CA, USA). A *p*‐value less than 0.05 was considered to signify statistical significance. ^*^ *p*<0.05, ^*^ ^*^ *p*<0.01, ^**^ ^*^ *p*<0.001, and ^****^ *p*<0.0001.

## Conflicts of Interest

The authors declare no conflicts of interest.

## Supporting information




**Supporting File**: advs74167‐sup‐0001‐SuppMat.docx.

## Data Availability

The data that support the findings of this study are available from the corresponding author upon reasonable request.
