# Supplementary material for: Intelligent Thermo‐Self‐Limited Magnetothermia with Heat‐Triggered TERT Silencing for Precision Synergetic Cancer Therapy
Source: Adv Sci (Weinh). 2026 Jan 30;13(20):e19168. doi: 10.1002/advs.202519168 (PMC13067769; doi:10.1002/advs.202519168)
Supplement: Supplementary file 1 — Supporting File: advs74167‐sup‐0001‐SuppMat.docx. [file ADVS-13-e19168-s001.docx]

**Supporting Information**

**Intelligent Thermo-Self-Limited Magnetothermia with Heat-Triggered TERT Silencing for Precision Synergetic Cancer Therapy**

*Liang Zhang, Mingfu Gong, Tao Sun, Shilin Xiao, Yue Zhao, Xiaofeng Yang, Wansu Zhang, Chunyu Zhou, Xu Liu, and Dong Zhang**

Department of Radiology, Xinqiao Hospital, Army Medical University, Chongqing 400037, P. R. China

*Corresponding Author

*Dong Zhang*, E-mail: hszhangd@tmmu.edu.cn.

Figure S1. Size analysis of IONPs in TEM image of Figure 1b.


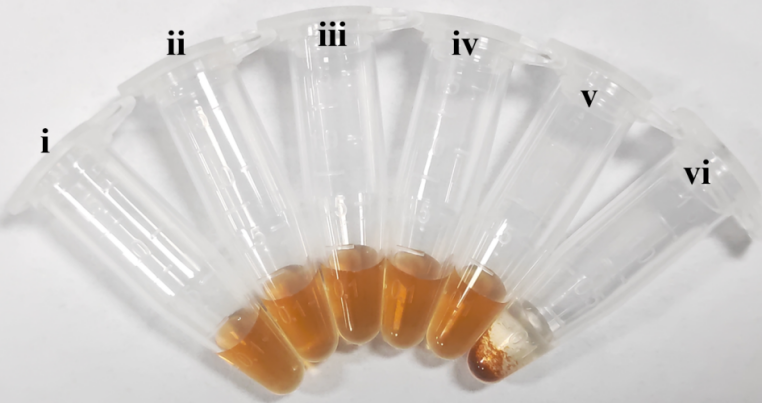


Figure S2. Specific aggregation of IONP@H_1_THs. i: Original IONP@H_1_THs; ii: IONP@H_1_THs+Random DNA1; iii: IONP@H_1_THs+Random DNA2; iv: IONP@H_1_THs+Random DNA3; v: IONP@H_1_THs+Random DNA4; vi: IONP@H_1_THs+Target TERT DNA.


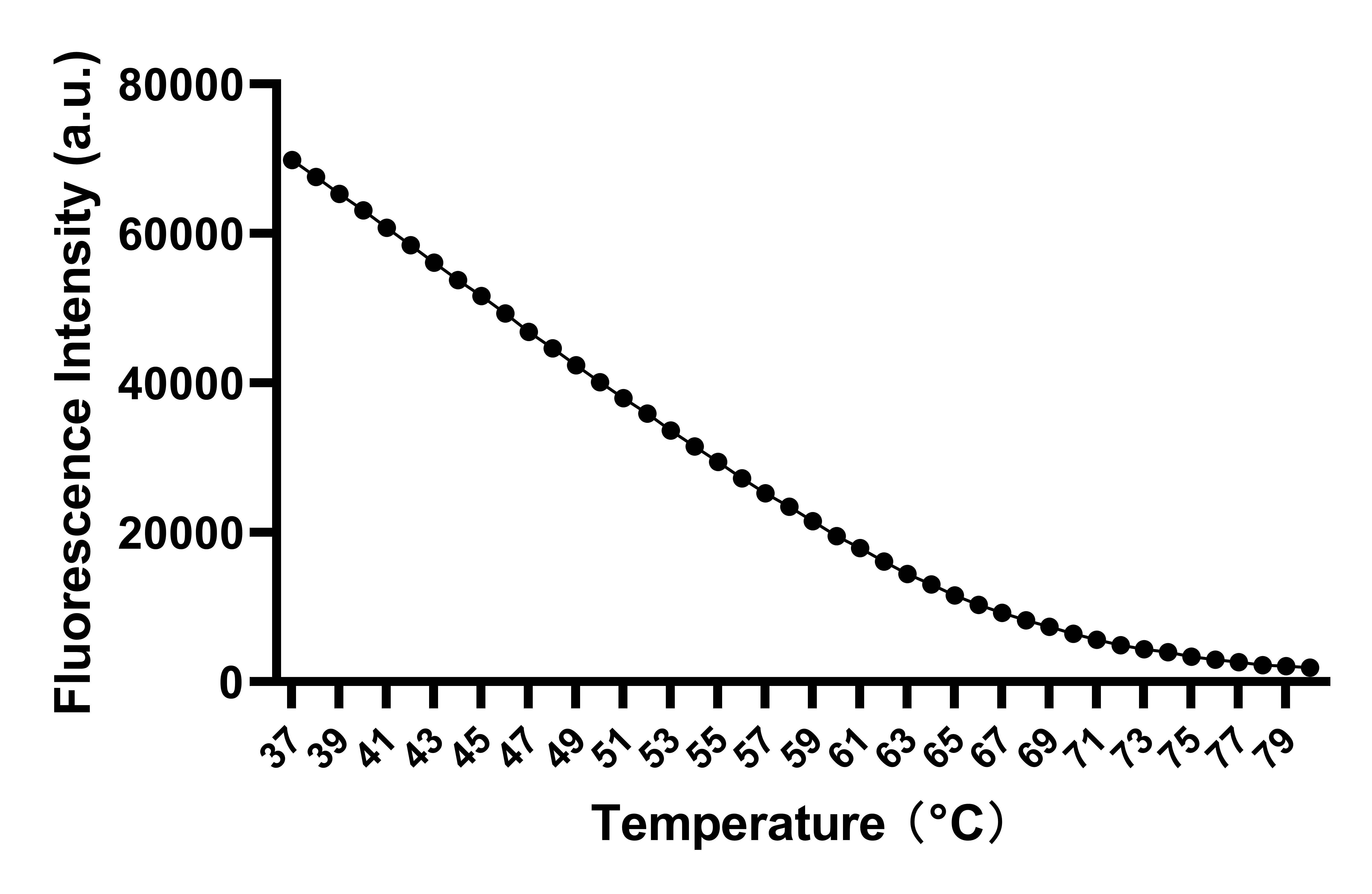


Figure S3. Fluorescence intensity-temperature curve to monitor dsDNA disassembly.


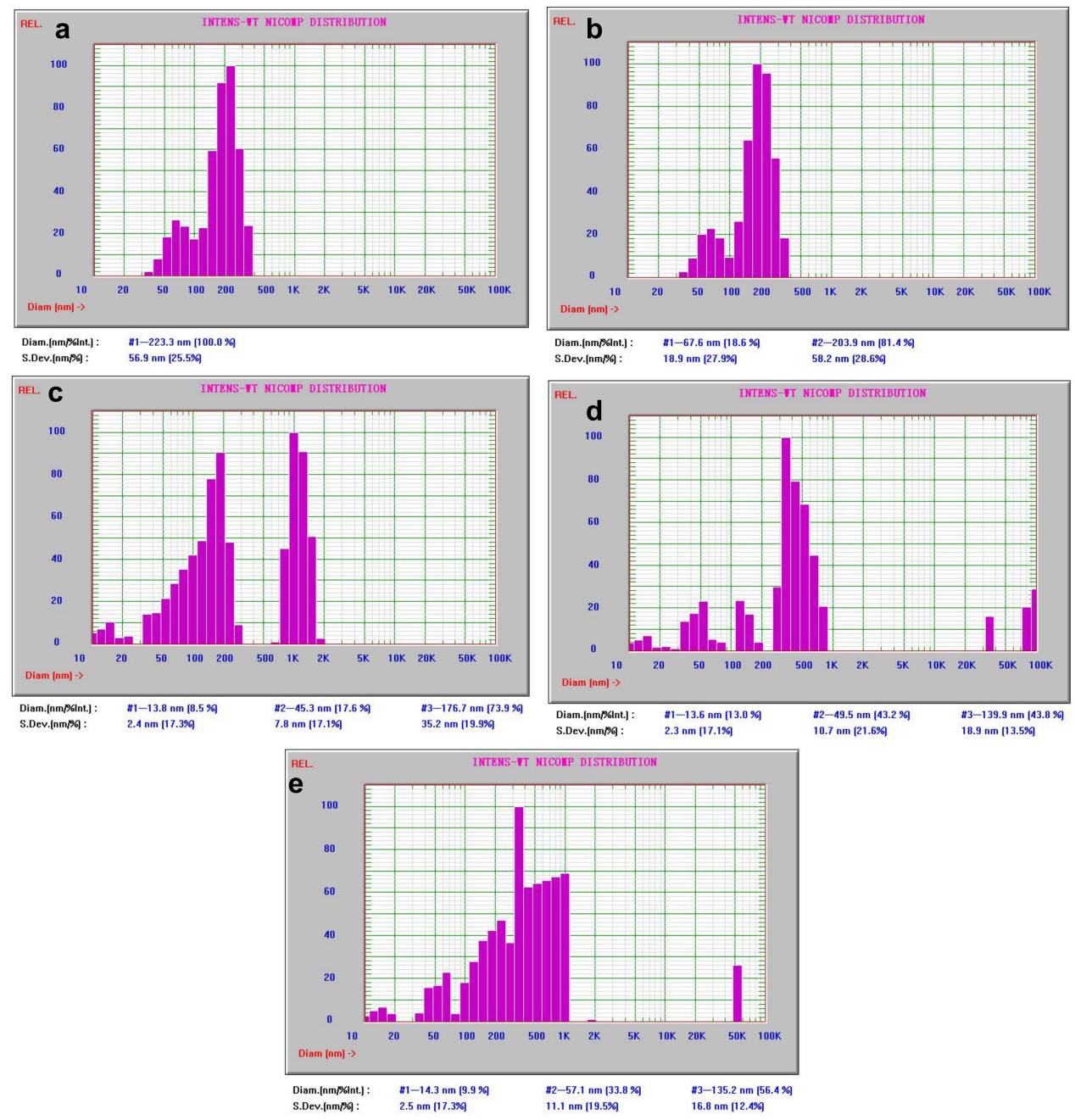


Figure S4. Hydrodynamic size of IONP@H_1_THs + Target after AMF (f = 345 kHz, field intensity = 13.1 kA/m) treatment. (a) Hydrodynamic size distribution of IONP@H_1_THs + Target at 37 °C. (b-d) Hydrodynamic size distribution of IONP@H_1_THs (500 μg/mL) + Target after AMF treated 5 min, 10 min, and 15 min, respectively. (e) Hydrodynamic size distribution of IONP@H_1_THs (400 μg/mL) + Target after AMF treated 15 min.


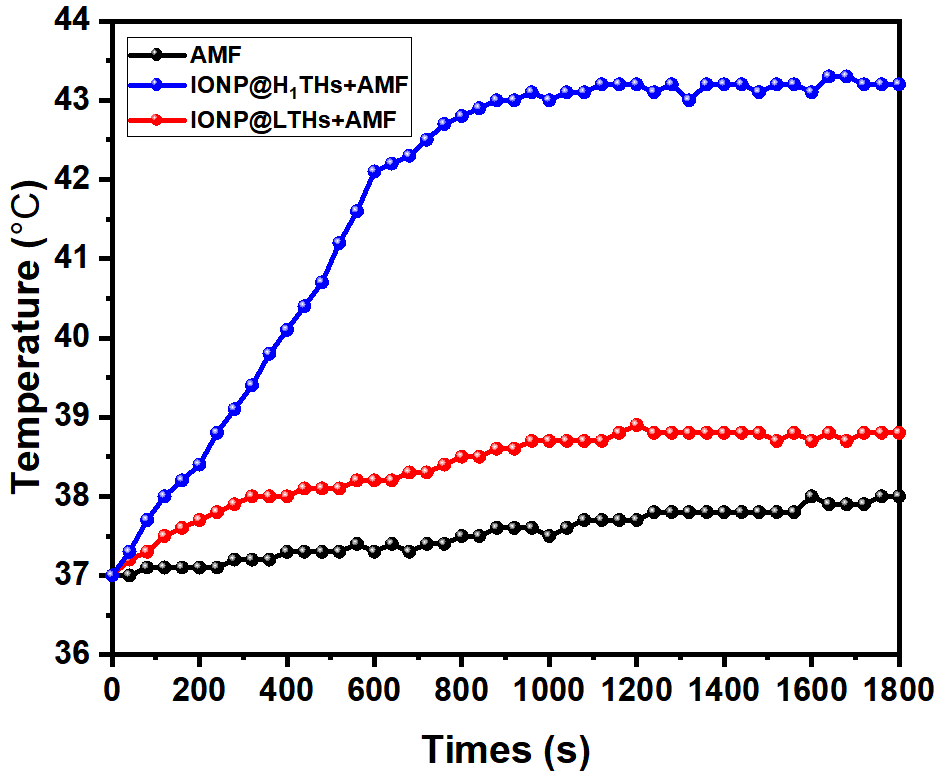


Figure S5. Time-temperature curve in cells after AMF (f = 345 kHz, field intensity = 13.1 kA/m) treatment.


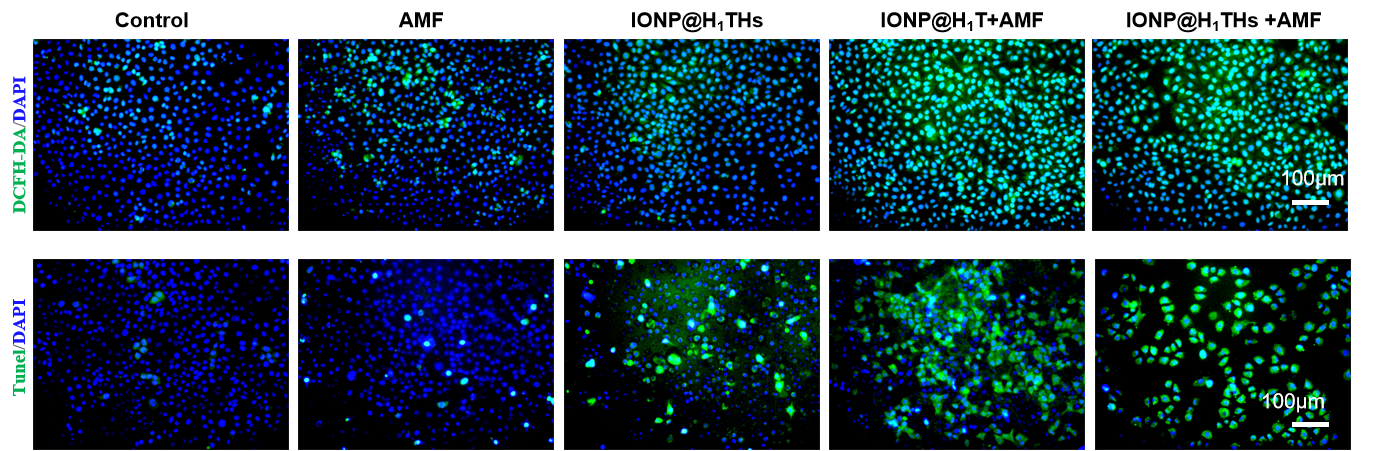


Figure S6. Analysis of reactive oxygen species and apoptosis in MDA-MB-231 cells after different treatments.


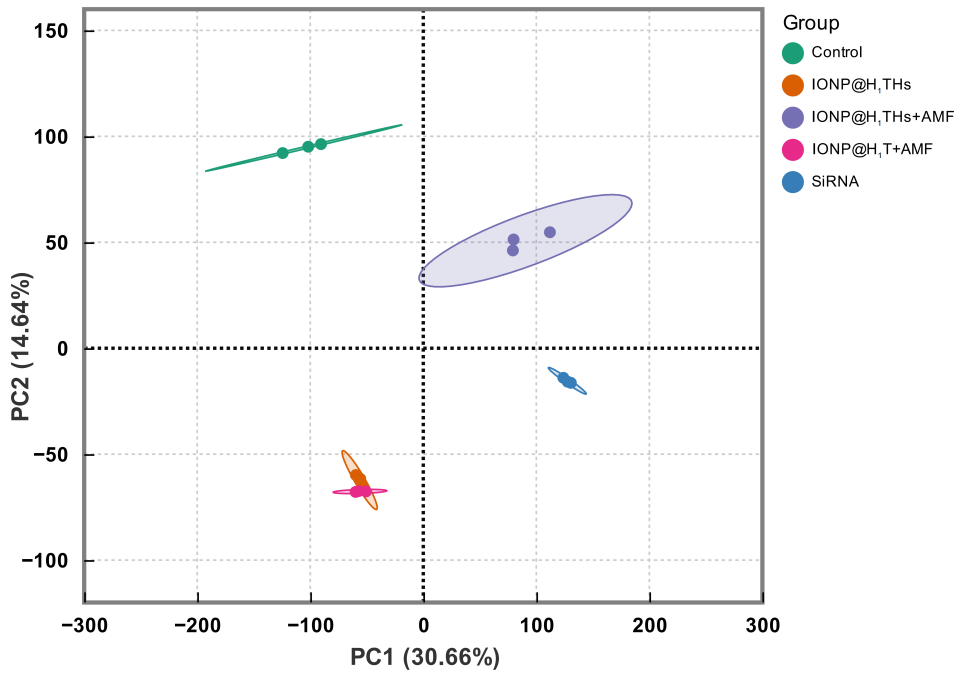


Figure S7. Principal component analysis of *RNA-Seq*.


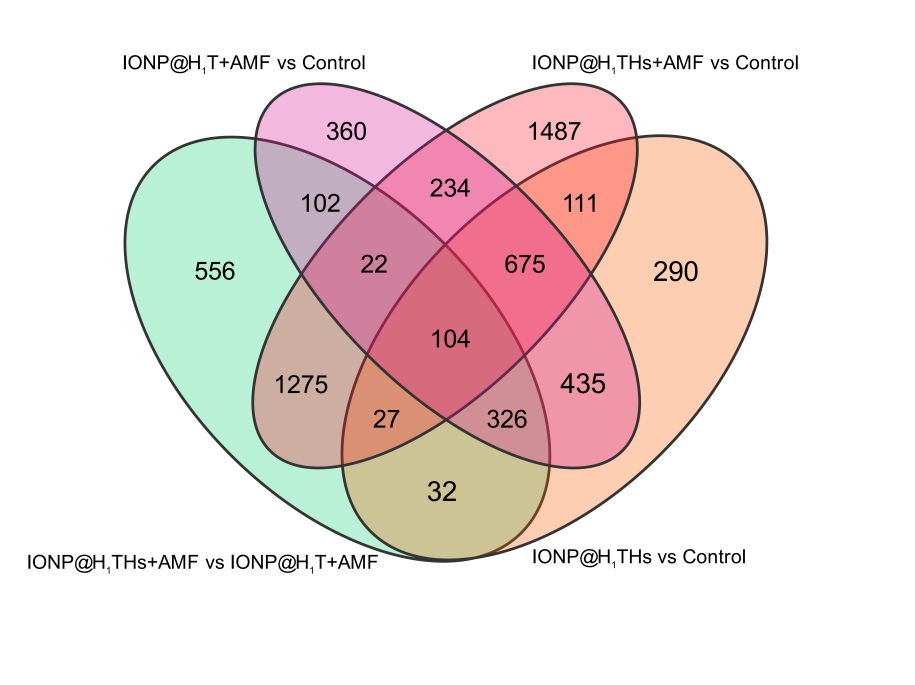


Figure S8. Venn diagram of four different comparisons.


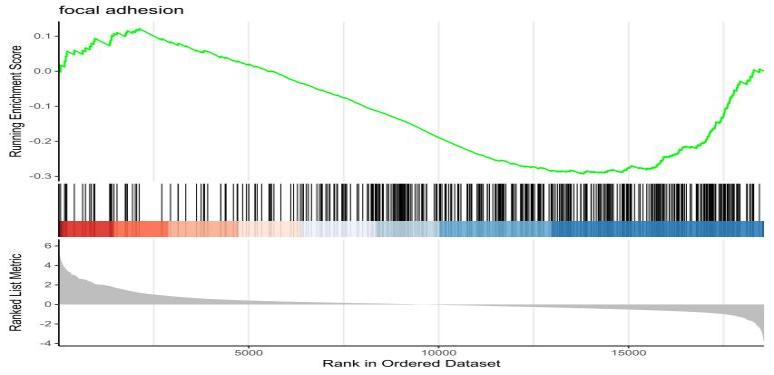


Figure S9. Gene Set Enrichment Analysis of focal adhesion between control and IONP@H_1_THs treated cells.


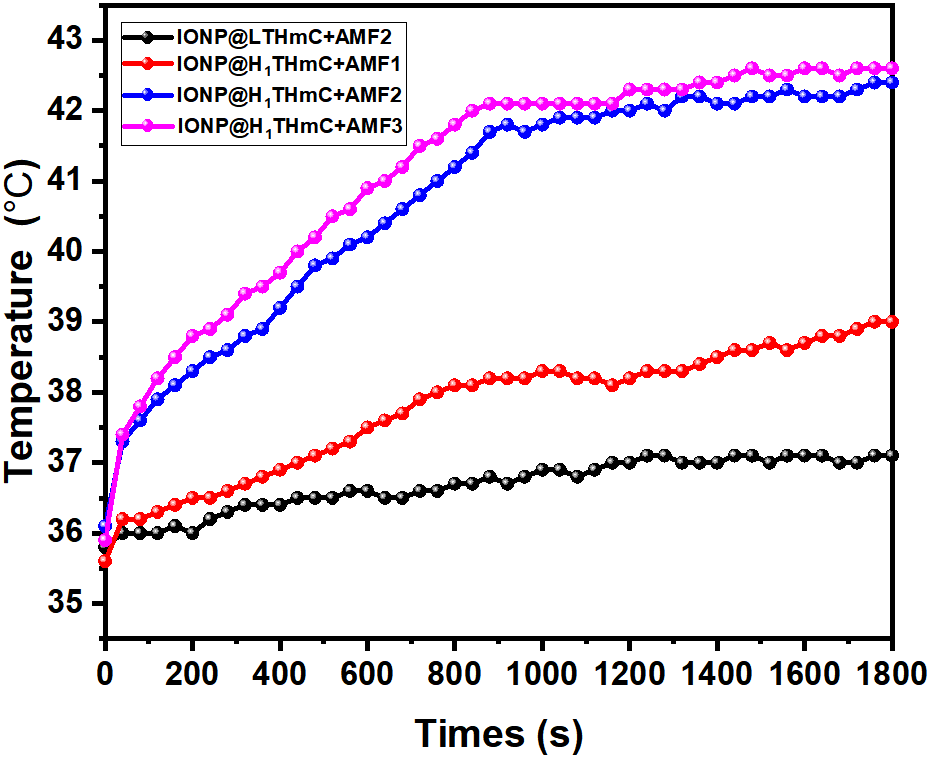


Figure S10. Temperature-time curve in tumors after different treatments.


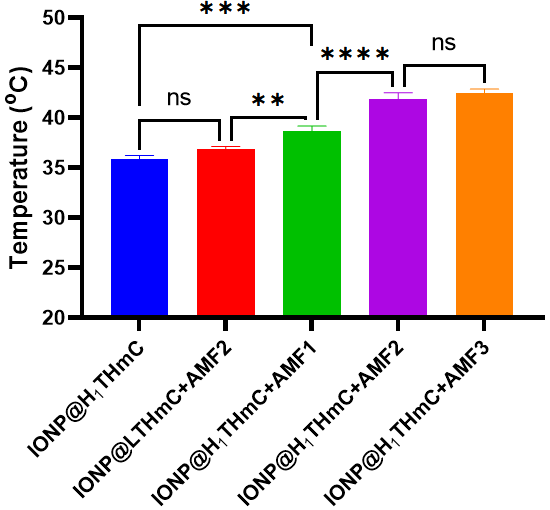


Figure S11. Local temperature of the tumors in mice after different treatments.





Figure S12. Biochemical analysis of liver and kidney functions at 1 day and 14 days after PBS, IONP@H_1_THs, or IONP@H_1_THs+AMF treatments.


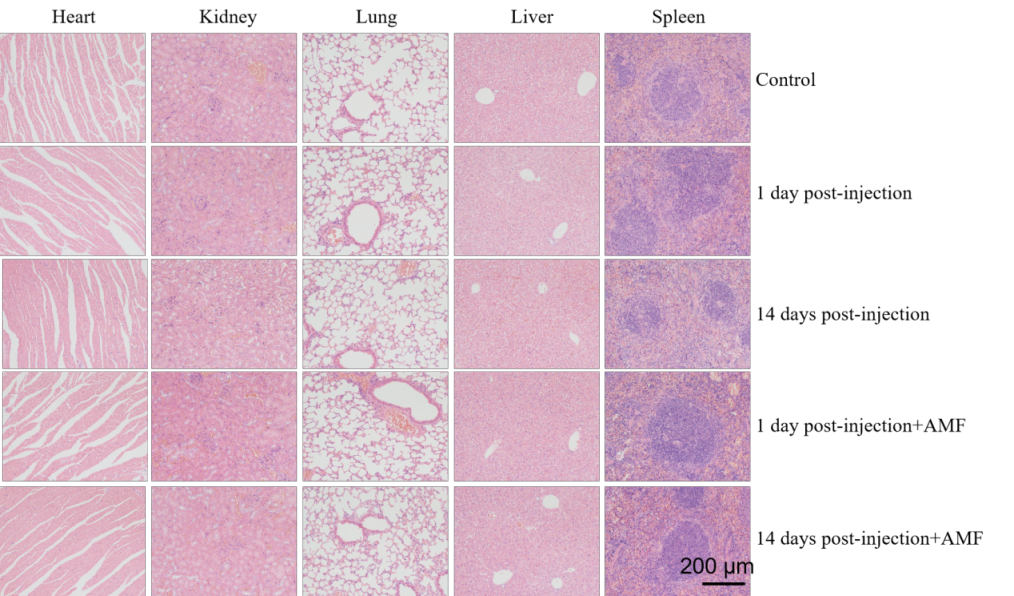


Figure S13. H&E images of major organs (Heart, Liver, Spleen, Lung, Kidney) at 1 day and 14 days after PBS, IONP@H_1_THs, or IONP@H_1_THs+AMF treatments.


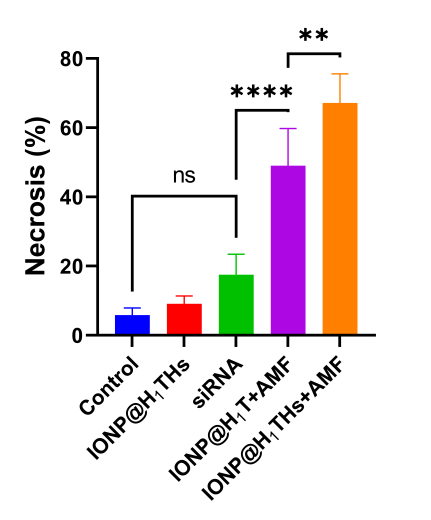


Figure S14. Quantitative analysis of the proportion of necrotic area in the results of H&E staining in Figure 7f.


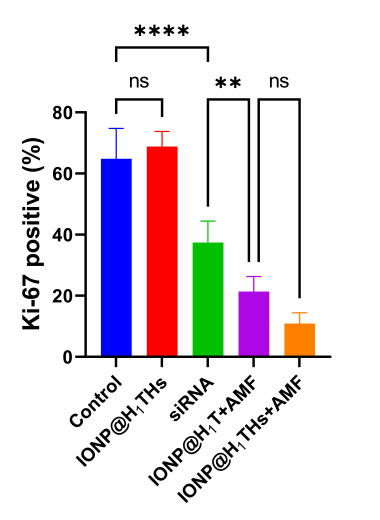


Figure S15. Quantitative analysis of the proportion of Ki-67 positive cells in Figure 7f.


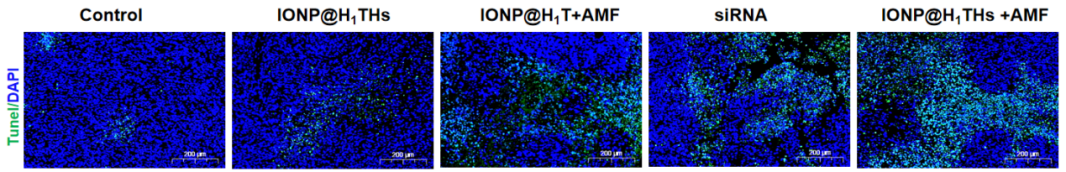


Figure S16. Fluorescence images of tumor sections after Tunel staining.


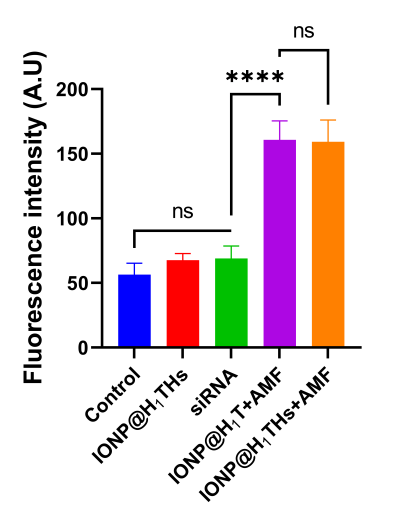


Figure S17. Quantitative analysis of the expression of Hsp70 in tumor tissue sections.


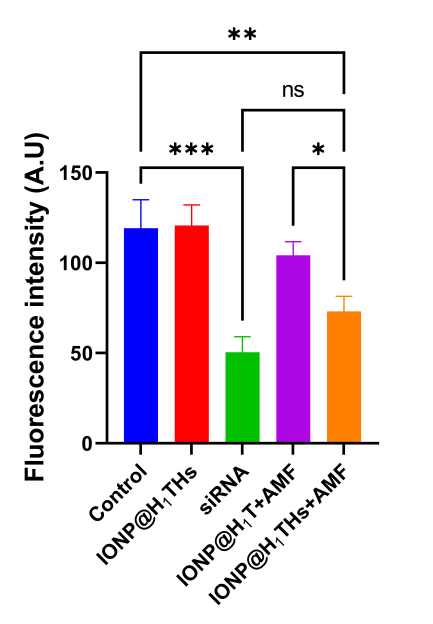


Figure S18. Quantitative analysis of the expression of TERT in tumor tissue sections.


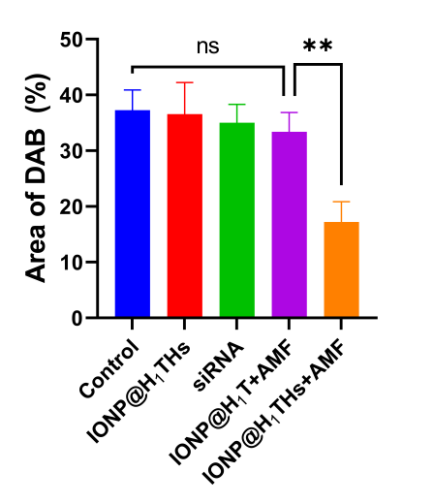


Figure S19. Quantitative analysis of the proportion of FAK positive area in Figure 7h.


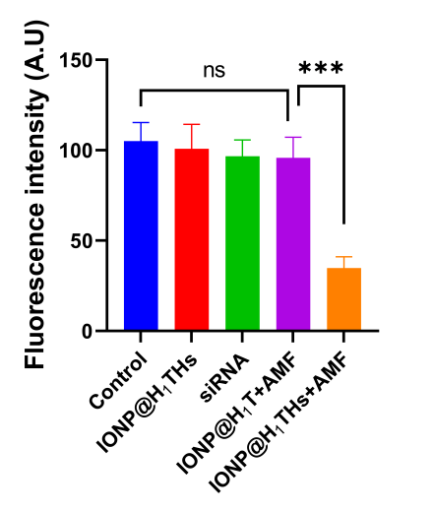


Figure S20. Quantitative analysis of the expression of Integrin in Figure 7h.


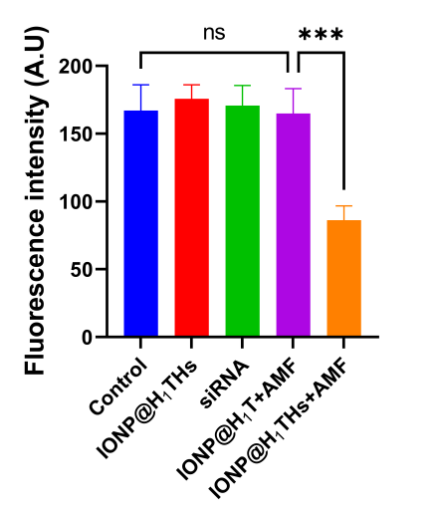


Figure S21. Quantitative analysis of the expression of RhoA in Figure 7h.


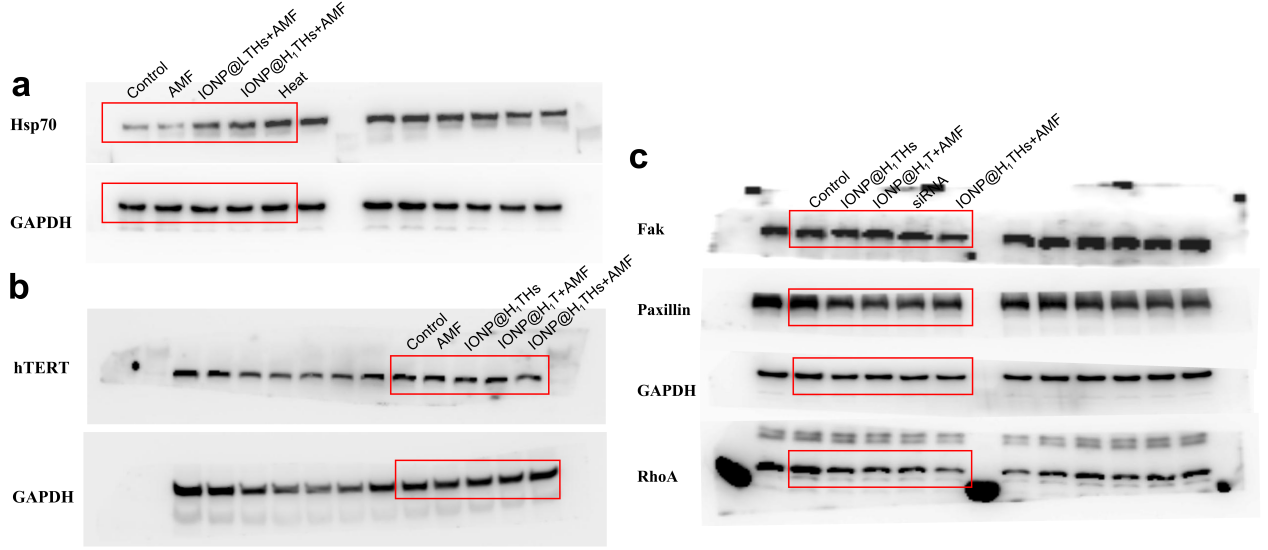


Figure S22. The original Western Blot images corresponding to Figure 3f (a), Figure 4e (b), and Figure 5d (c).

Table S1. Nucleotide sequences used in this study

| Name | Sequence (5'-3') |
| --- | --- |
| H1-DNA (46.8) | COOH-gggggCCATCAGAGCC*AGTCTCATGAGACT* |
| H2-DNA (38.6) | COOH-gggggCCATCAGAGCCAG*TCTCTTATAAGAGA* |
| H3-DNA (50.1) | COOH-gggggCCATCAGAGCC*AGTCTCAATTGAGACT* |
| L-DNA | COOH-gggggCCATCAGAGCCAGTCTCTT |
| TR-DNA | GGTGGTGGTGGTTGTGGTGGTGGTGG-ttttttttttttttttt-*GAAGCCGCGGTTGAAGG*TGAGACTGGCTCTGATGGCCCCC (Aptamer-linker-Responsive) |
| HSP70 promoter mediated Functional DNA | CCTTCAACCGCGGCTTC-HSP70 promoter-shRNA/mCherry-(Reverse complementary DNA strand of HSP70 promoter-shRNA/mCherry) |
| HSP70 promoter | AACCCCTGGAATATTCCCGACCTGGCAGCCTCATCGAGCTCGGTGATTGGCTCAGAAGGGAAAAGGCGGGTCTCCGTGACGACTTATAAAAGCCCAGGGGCAAGCGGTCCGGA |
| mCherry | ATGGTGAGCAAGGGCGAGGAGGATAACATGGCCATCATCAAGGAGTTCATGCGCTTCAAGGTGCACATGGAGGGCTCCGTGAACGGCCACGAGTTCGAGATCGAGGGCGAGGGCGAGGGCCGCCCCTACGAGGGCACCCAGACCGCCAAGCTGAAGGTGACCAAGGGTGGCCCCCTGCCCTTCGCCTGGGACATCCTGTCCCCTCAGTTCATGTACGGCTCCAAGGCCTACGTGAAGCACCCCGCCGACATCCCCGACTACTTGAAGCTGTCCTTCCCCGAGGGCTTCAAGTGGGAGCGCGTGATGAACTTCGAGGACGGCGGCGTGGTGACCGTGACCCAGGACTCCTCCCTGCAGGACGGCGAGTTCATCTACAAGGTGAAGCTGCGCGGCACCAACTTCCCCTCCGACGGCCCCGTAATGCAGAAGAAGACCATGGGCTGGGAGGCCTCCTCCGAGCGGATGTACCCCGAGGACGGCGCCCTGAAGGGCGAGATCAAGCAGAGGCTGAAGCTGAAGGACGGCGGCCACTACGACGCTGAGGTCAAGACCACCTACAAGGCCAAGAAGCCCGTGCAGCTGCCCGGCGCCTACAACGTCAACATCAAGTTGGACATCACCTCCCACAACGAGGACTACACCATCGTGGAACAGTACGAACGCGCCGAGGGCCGCCACTCCACCGGCGGCATGGACGAGCTGTACAAGTAA |
| TERT shRNA | GAAGAGTGTCTGGAGCAAGTT*CTCGAG*AACTTGCTCCAGACACTCTTC (Sense-*loop*-anti-Sense chain) |
